# A molecular portrait of epithelial–mesenchymal plasticity in prostate cancer associated with clinical outcome

**DOI:** 10.1038/s41388-018-0488-5

**Published:** 2018-09-07

**Authors:** Nataly Stylianou, Melanie L. Lehman, Chenwei Wang, Atefeh Taherian Fard, Anja Rockstroh, Ladan Fazli, Lidija Jovanovic, Micheal Ward, Martin C. Sadowski, Abhishek S. Kashyap, Ralph Buttyan, Martin E. Gleave, Thomas F. Westbrook, Elizabeth D. Williams, Jennifer H. Gunter, Colleen C. Nelson, Brett G. Hollier

**Affiliations:** 1 Australian Prostate Cancer Research Centre—Queensland, Institute of Health and Biomedical Innovation, Faculty of Health, School of Biomedical Sciences, Queensland University of Technology, Princess Alexandra Hospital, Translational Research Institute, Brisbane, QLD Australia; 2 Vancouver Prostate Centre, Department of Urologic Sciences, University of British Columbia, Vancouver, Canada; 3 Glycation and Diabetic Complications Group, Mater Research Institute, Translational Research Institute, School of Medicine, University of Queensland, Brisbane, QLD Australia; 4 Tissue Repair and Regeneration Program, Institute of Health and Biomedical Innovation, Queensland University of Technology, Brisbane, QLD Australia; 5 Verna and Marrs McLean Department of Biochemistry and Molecular Biology, Baylor College of Medicine, Houston, TX USA

**Keywords:** Prostate cancer, Metastasis

## Abstract

The propensity of cancer cells to transition between epithelial and mesenchymal phenotypic states via the epithelial–mesenchymal transition (EMT) program can regulate metastatic processes, cancer progression, and treatment resistance. Transcriptional investigations using reversible models of EMT, revealed the mesenchymal-to-epithelial reverting transition (MErT) to be enriched in clinical samples of metastatic castrate resistant prostate cancer (mCRPC). From this enrichment, a metastasis-derived gene signature was identified that predicted more rapid cancer relapse and reduced survival across multiple human carcinoma types. Additionally, the transcriptional profile of MErT is not a simple mirror image of EMT as tumour cells retain a transcriptional “memory” following a reversible EMT. This memory was also enriched in mCRPC samples. Cumulatively, our studies reveal the transcriptional profile of epithelial–mesenchymal plasticity and highlight the unique transcriptional properties of MErT. Furthermore, our findings provide evidence to support the association of epithelial plasticity with poor clinical outcomes in multiple human carcinoma types.

## Introduction

Metastatic tumour burden is the major cause of cancer-related mortality in carcinomas arising in the epithelia of organs such as the breast, colon, and prostate [1–3]. In the prostate, androgens promote the differentiation, proliferation, and survival of benign and malignant tissue to drive castrate-resistant progression. These biological consequences are mediated through the androgen receptor (AR), which leads to the transcriptional activation and repression of multiple gene networks. Androgen/AR targeted therapies (ATTs) exploit this dependence in advanced prostate cancer (PCa) patients, and may involve parallel or sequential use of differential androgen synthesis or AR inhibitors. While these therapies delay clinical progression and extend survival, they are not curative. Despite the addition of potent ATTs such as abiraterone (Zytiga®), and enzalutamide (Xtandi®) in 2nd and 3rd line therapies, there is still an inevitable progression to castrate-resistant prostate cancer (CRPC) [4].

With the use of ATTs, the differentiation pressure of androgens is lost, setting up an environment that fosters castrate-resistant cell dedifferentiation and trans-differentiation (collectively termed castrate-resistant plasticity). This adaptive plasticity is thought to allow PCa cells to transition between phenotypic states and survive ATTs, promoting castrate-resistant recurrence and metastasis. Recent evidence implicates the epithelial-to-mesenchymal transition (EMT) as a defined plasticity program potentiated by inhibition of the androgen axis [5]. Studies have shown that the AR directly represses the expression of the EMT-inducing transcription factors (EMT-TFs), such as SNAI1/Snail, by binding to specific AR-responsive elements within its promoter [6]. Snail can also promote enzalutamide resistance in sensitive PCa cells [7] and has been reported to correlate with aggressive disease and metastasis [7]. Thus, dysregulation of AR signalling via ATTs such as enzalutamide can promote EMT and therapy resistance [6].

The transition to a mesenchymal phenotype via EMT endows carcinoma cells with invasive traits that facilitate metastasis [8]. Studies in preclinical models indicate that the transient induction of EMT and subsequent reversion of castrate-resistant cells to their epithelial phenotype (termed as the mesenchymal–epithelial reverting transition—MErT) is required for the formation of overt metastases [9–12]. This oscillation between epithelial and mesenchymal states represents a dynamic and complex set of events that contribute, and are perhaps paramount, to successful metastatic dissemination and colonisation. Despite this, very few studies have investigated the cycling of this plasticity in malignancy and its lasting impact on castrate resistant cell behaviour and treatment resistance. This stems in part from the lack of robust preclinical models that allow for precisely controlled studies of EMT and MErT within isogenic cancer cell populations. Furthermore, in a cancer setting, there has been an unchallenged perception that MErT is the mere antithesis of EMT. As a result, there is limited direct evidence in clinical samples for the relevance and impact of this dynamic epithelial–mesenchymal castrate-resistant cell plasticity to clinical metastasis, treatment resistance, and patient prognosis.

To address this, our study describes the establishment of PCa models driven by the inducible and reversible expression of the transcription factors SNAI1/Snail or SNAI2/Slug. Transcriptional profiling of PCa cells oscillating between EMT and MErT states has identified the dynamic events underpinning these transitions to reveal a molecular portrait of castrate-resistant cell epithelial–mesenchymal plasticity (EMP). Interrogation of gene expression profiling from patient datasets identified a transcriptional signature that is enriched in clinical samples of metastatic CRPC (mCRPC), which was also observed to associate with poor patient prognosis when enriched in primary castrate-resistant samples. Finally, we provide provocative data that castrate-resistant cells retain a transcriptional “memory” from experiencing castrate-resistant EMP, and retain stable alterations in transcript expression also found to be enriched in clinical samples of mCRPC.

## Results

### Reversible expression of snail accurately recapitulates biological phenotypes associated with EMT/MErT

**Fig. 1:**
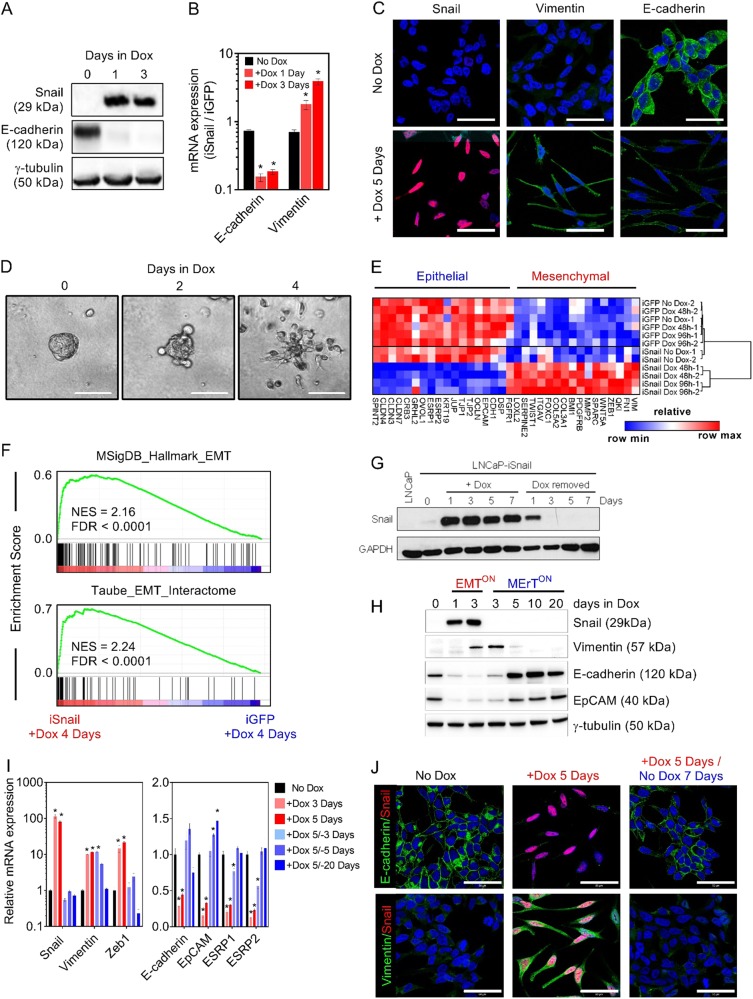
The LNCaP–iSnail model is a dynamic model of epithelial–mesenchymal plasticity. **a** Western blot showing protein expression for Snail and E-cadherin in untreated LNCaP–iSnail cells, and cells treated with Dox for 1 and 3 days. γ-Tubulin was visualised for loading control purposes. **b** Gene expression of E-cadherin and vimentin LNCaP–iSnail cells treated with Dox for 1 and 3 days. Gene expression was normalised to *RPL32*. Fold change is relative to the respective LNCaP–iGFP cells treated with Dox for 1 and 3 days. One-way ANOVA, *p < 0.05. Error bars indicate SEM for biological triplicates. **c** Representative immunofluorescence images showing expression of Snail, vimentin, and E-cadherin in untreated LNCaP–iSnail cells and treated with Dox for 5 days. Nuclei were visualised with DAPI. Scale bars = 50 µm. **d** Phase contrast images of LNCaP–iSnail cells grown as multicellular spheroids in a Matrigel™ assay prior to treatment (Day 0) and treated with Dox for 2 and 4 days. Scale bars = 100 µm. **e** Heatmap showing the expression of known epithelial and mesenchymal genes from untreated LNCaP–iSnail and LNCaP–iGFP spheroids, and treated with Dox for 2 and 4 days. Heatmap color refers to normalised *z*-score. **f** GSEA enrichment plots of established EMT signatures [14, 20] within the transcriptional program regulated by 4 days of Snail-induction, compared to Dox-treated LNCaP–iGFP cells. **g** Western blot showing Snail protein levels in untreated parental LNCaP cells, untreated LNCaP–iSnail cells, and LNCaP–iSnail cells treated with Dox for 7 days followed by removal for 7 days. GAPDH protein was visualised for loading control purposes. **h** Western blot showing expression of Snail, vimentin, E-cadherin, and EpCAM proteins in untreated LNCaP–iSnail cells, and treated with Dox for 1 and 3 days followed by removal for 3, 5, 10, and 20 days. γ-Tubulin was visualised for loading control purposes. **i** Gene expression of *Snail*, *vimentin*, *Zeb1, E-cadherin*, *EpCAM*, *ESRP1*, and *ESRP2* in LNCaP–iSnail cells treated with Dox for 3 and 5 days, followed by removal for 3, 5, and 20 days. Gene expression was normalised to *RPL32*. Fold change is relative to untreated cells. One-way ANOVA, *p < 0.05. Error bars indicate SEM for biological triplicates. **j** Representative immunofluorescence images of E-cadherin and vimentin proteins in untreated LNCaP–iSnail cells, and treated with dox for 5 days followed by removal for 7 days. Nuclei were visualised using DAPI. Scale bars = 50 µm

To generate a reversible EMT system, we recombined the cDNA of the master EMT-TF, SNAI1 (Snail), into the doxycycline (Dox)-inducible pINDUCER20 lentiviral expression construct [13]. The pINDUCER20-Snail construct was then transduced into the androgen-dependent human PCa cell line, LNCaP (LNCaP–iSnail). The LNCaP cell line was chosen as it exhibits secretory epithelial characteristics and closely mimics androgen-sensitive PCa. The treatment of LNCaP–iSnail cells with Dox induced robust Snail protein expression (Fig. 1a–c), triggering a reduction in E-cadherin (Fig. 1a–c) and increased vimentin expression (Fig. 1b, c). This was accompanied by morphological alterations characteristic of a mesenchymal phenotype (Fig. 1c). This effect was absent in LNCaP cells generated to have Dox-inducible expression of green fluorescent protein (GFP) (LNCaP–iGFP; Figure S1). In contrast to constitutive expression systems, the inducible nature of Snail expression made it possible to visualise and assess the switch of preformed epithelial tumour spheroids into a highly invasive phenotype following Snail expression (Fig. 1d, Figure S2; Supplementary video 1 and 2). Microarray analysis of these tumour spheroids allowed us to examine the timing of key transcriptional events during the EMT-mediated invasive switch (Fig. 1e). Using gene set enrichment analysis (GSEA), the transcriptional program activated in LNCaP–iSnail tumour spheroids by Dox treatment (as compared to LNCaP–iGFP tumour spheroids) was validated to be enriched in established transcriptional signatures of EMT (Fig. 1f).

We then examined the reversibility of EMT in our system. Removal of Dox from treated LNCaP–iSnail cells resulted in the cessation of Snail expression (Fig. 1g), followed by the re-expression of epithelial proteins, E-cadherin and EpCAM (Fig. 1h, i). This was paralleled by a, albeit slower, decrease in the mesenchymal marker vimentin expression (Fig. 1h, i). The loss of mesenchymal markers (*VIM* and *ZEB1*) and restoration of epithelial gene expression (*CDH1*, *EPCAM*, *ESRP1*, and *ESRP2*) (Fig. 1i) was accompanied with the return to an epithelial-like morphology (Fig. 1j). Taken together, these results support the LNCaP–iSnail model as a dynamic model of EMP, allowing for the tight regulation and reversible transition of cells between epithelial and mesenchymal phenotypic states.

### The transcriptional landscape of epithelial–mesenchymal plasticity is temporally dynamic

**Fig. 2:**
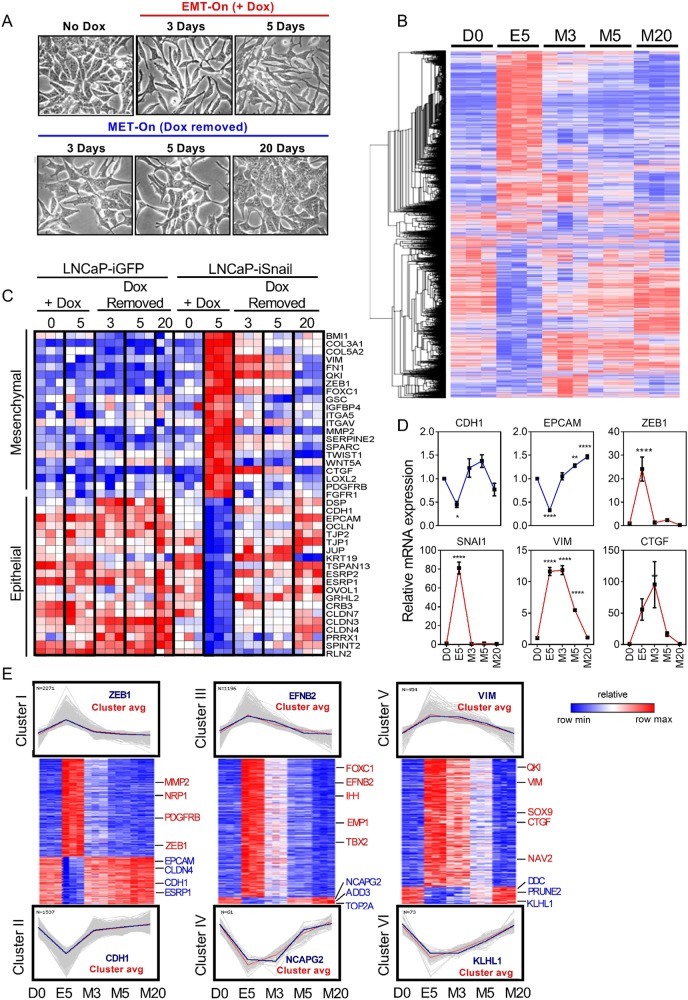
An overview of the transcriptional landscape of Snail-regulated EMP. **a** Phase-contrast images of LNCaP–iSnail cells treated with Dox for 5 days to induce EMT and subsequently removed to allow for MErT over 20 days. **b** Heatmap representation showing the expression of genes altered at any time during the 5 day EMT and 3, 5, and 20 days of MErT (5157 gene identities can be found in the supplementary dataset 1). **c** Heatmap representation showing the expression of known epithelial and mesenchymal genes in LNCaP–iSnail and LNCaP–iGFP cells treated with Dox for 5 days followed by removal for 3, 5, and 20 days. **d** qRTPCR validation of *CDH1*, *EpCAM*, *ZEB1*, *SNAI1*, *VIM*, and *CTGF*. Gene expression was normalised to *RPL32*. Fold change is relative to untreated LNCaP–iSnail cells (NoDox). One-way ANOVA, *p < 0.05. Error bars indicate SEM for biological triplicates. **e** Six transcriptional clusters were generated through pattern matching of select genes (blue line) and classified by temporal reversion pattern. Clusters I and II show reverting expression by day 3 of MErT (MErT3; M3); Clusters III and IV by day 5 (MErT5; M5); and Clusters V and VI by day 20 (MErT20; M20). Cluster avg is the average transcriptional pattern of the transcript cluster. Heatmap representation showing the gene expression of the genes within each of the three cluster pairs. Heatmap color for all heatmaps refers to normalised *z*-score

The reversibility of a Snail-mediated EMT made it possible to characterize a timeline of transcriptional changes integral to MErT. LNCaP–iSnail cells were treated with Dox for 5 days to induce EMT, followed by removal for up to 20 days to allow cells sufficient time to revert back to an epithelial phenotype (Fig. 2a). Microarray-based gene expression profiling was performed on untreated LNCaP–iSnail cells (D0), cells treated with Dox for 5 days (EMT; E5), and cells that had Dox removed for 3, 5, and 20 days (MErT; M3, M5, M20). For control purposes, the same profiling was conducted using the LNCaP–iGFP cells. The microarray data were refined by removing probes that became differentially expressed in the LNCaP–iGFP cells at any timepoint during the experiment (fold change ± 1.5, *p* *≤* 0.05; for more information see Materials and methods section). The resulting refined dataset is subsequently referred to as the “Snail-dataset” (36,931 probes, which collapsed to 12,223 genes, supplementary dataset 1).

In the first instance, we focused on the transcriptional changes evoked by an EMT (E5 vs D0, fold change ± 1.5, *p* *≤* 0.05; 13,425 probes collapsed to 5157 genes; Fig. 2b; Supplementary dataset 1). Examination of the expression pattern of established epithelial and mesenchymal markers confirmed the successful induction of EMT and subsequent MErT (as compared to the transcriptional levels in LNCaP–iGFP cells at corresponding time points) (Fig. 2c). The expression levels of *CDH1 EPCAM*, *ZEB1*, *SNAI1*, *VIM*, and *CTGF* were also validated via qRT-PCR (Fig. 2d). Closer inspection of the transcriptional patterns occurring during MErT revealed the transient nature of key EMT markers. While the majority of established epithelial and mesenchymal markers returned to pre-EMT baseline levels within 3 days of MErT (eg. *ZEB1* and *CDH1*), subsets of transcripts were found to have slower kinetics and required up to 20 days to return to baseline (e.g. *VIM*, *CTGF*) (Fig. 2c, d).

We identified six discrete transcriptional clusters that operated during MErT (Fig. 2e). Clusters I and II contained transcripts reverting to baseline levels early (3 days of MErT; cluster I, *n* = 1208 transcripts; cluster II, *n* = 572 transcripts); clusters III and IV contained transcripts reverting with intermediate kinetics to baseline (5 days of MErT; cluster III, *n* = 712 transcripts; cluster IV, *n* = 33 transcripts); and clusters V and VI contained transcripts with a slower reversion to baseline levels (20 days of MErT; cluster V, *n* = 269 transcripts; cluster VI, *n* = 36 transcripts) (for cluster transcript identities see supplementary dataset 1). GSEA of the clusters combined revealed enrichment of “Hallmark” gene sets (from the Molecular Signatures Database; MSigDB; Broad Institute, MIT [14]) for cell cycle (E2F targets, G2M checkpoint) and metabolism (oxidative phosphorylation) (Figure S3). GSEA performed on the individual clusters revealed that the enrichment of these biological signatures was more prominent in the early MErT clusters (clusters I and II) as compared to the later stages of MErT (clusters III–VI; Figure S3). Taken together, these findings provide the first molecular portrait of the dynamic and reversible transcriptional alterations occurring as tumour cells cycle between EMT and MErT-like states.

### Identification and validation key biological processes operating during epithelial–mesenchymal-plasticity

**Fig. 3:**
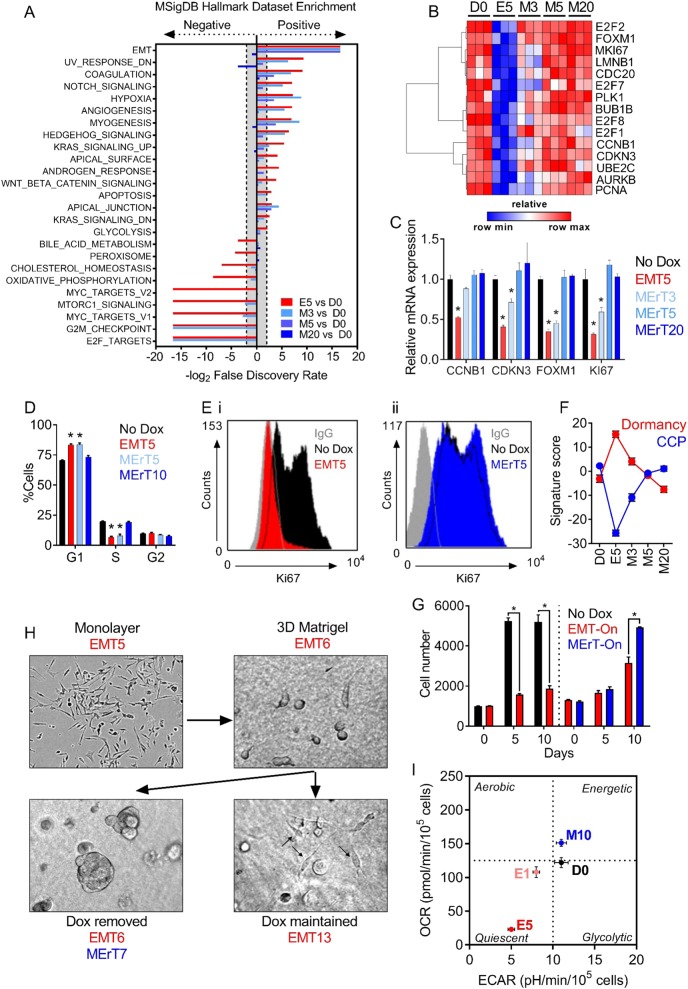
Snail-regulated EMP influences cell cycle and metabolic activity in prostate cancer cells. **a** Enrichment of indicated hallmark datasets from the MSigDB [14] during the EMT/MErT timecourse. **b** Heatmap showing the expression of selected cell cycle-related genes in untreated LNCaP–iSnail cells (No Dox), cells treated with dox for 5 days (EMT5; E5), and subsequently removed for 3, 5, and 20 days (MErT3-20). Heatmap color refers to normalised *z*-score. **c** Gene expression of *CCNB1*, *CDKN3*, *FOXM1*, and *MKI67* in LNCaP–iSnail cells treated with dox for 5 days (EMT5), and subsequently removed for 3, 5, and 20 days (MErT3-20). Gene expression was normalised to *RPL32*. Fold change is relative to untreated LNCaP–iSnail cells (NoDox). One-way ANOVA, *p < 0.05. Error bars indicate SEM for biological triplicates. **d** Flow cytometry cell cycle analysis of untreated LNCaP–iSnail and LNCaP–iGFP cells, and cells treated with dox for 5 days (EMT5) and subsequently removed for 5 and 10 days (MErT5–10). One-way ANOVA, *p < 0.05. Error bars indicate SEM for biological triplicates. **e** Histogram representation of flow cytometry analysis for the expression of Ki-67 antigen surface expression in untreated LNCaP–iSnail cells (D0), and cells treated with dox for 5 days (EMT5), followed by removal for 5 days (MErT5). Grey histogram shows isotype control (IgG). **f** Enrichment score of previously published signatures of cell cycle progression (CCP) [15] and tumour dormancy [16] in untreated LNCaP–iSnail cells (NoDox), and cells treated with dox for 5 days (EMT5) followed by removal for 3, 5, and 20 days (MErT3–20). **g** Left of dotted line: raw cell number of untreated LNCaP–iSnail cells at 5 and 10 days (No Dox), and cells treated with Dox for 5 and 10 days (EMT-On). Right of dotted line: LNCaP–iSnail cells were treated with Dox for 5 days, re-seeded into new plates, and cell number quantified after an additional 5 and 10 days of Dox treatment (EMT-On) or had Dox removed (MErT-On). One-way ANOVA, *p < 0.0001. Error bars indicate SEM for biological triplicates. **h** Representative images of LNCaP–iSnail cells treated with Dox for 5 days in a monolayer (top left) prior to seeding as single cells in 3D Matrigel™ assays (top right). LNCaP–iSnail cells were then either maintained in Dox (bottom left) or had Dox removed (bottom right) for an additional 7 days. **i** Phenogram showing the relative metabolic state of untreated LNCaP–iSnail cells (D0), treated with Dox for 1 and 5 days (E1–E5), and removed for 10 days (M10). *x*-Axis portrays the basal oxygen consumption rate (OCR) and *y*-axis the extracellular acidification rate (ECAR)

To gain insights on the global biological processes regulated within the transcriptional alterations taking place during the EMT and MErT states, we performed GSEA on the Snail-dataset using the “Hallmark” gene collection within the MSigDB (Fig. 3a). The transcriptional alterations evoked as cells transitioned from their parental epithelial state to a Snail-induced mesenchymal state, were positively enriched in hallmark signatures such as EMT, coagulation, Notch signalling, and hypoxia (Fig. 3a). In parallel, a negative enrichment for signatures related to cell cycle and metabolism, including E2F Targets, G2/M Checkpoint, and Oxidative Phosphorylation was observed (Fig. 3a). The reversion of Snail-induced mesenchymal cells (E5) via a 20 day MErT (M20), led to a transcriptional reprogramming of cells back to an epithelial-like phenotype similar to that of non-induced cells (Fig. 3a).

In support of these findings, EMT resulted in the transcriptional downregulation of key mediators of cell cycle progression, including E2F Targets (*CDKN3*, *MKI67*, and *PCNA*) and G2/M checkpoint regulators (*CCNB1*, *FOXM1*, and *CDC20*) (Fig. 3b), whereby *CCNB1*, *CDKN3*, *FOXM1*, and *MKI67* were further validated via qRT-PCR (Fig. 3c). Basal expression of these cell cycle regulators was restored by 20 days of MErT (MErT20; Fig. 3c) as tumour cells reacquired their epithelial phenotype. Downstream functional assessment of these alterations confirmed EMT-induced cells to be in G0/G1-phase arrest (Fig. 3d) with a substantially reduced population of proliferating antigen-Ki67 expressing cells (Fig. 3ei). Release from the mesenchymal state with a MErT restored both the cell cycle and Ki67 profile of cells to the pre-EMT epithelial state (Fig. 3d, eii). Comparative examination of previously reported tumour-derived signatures of cell cycle progression (CCP) [15] and dormancy [16], revealed MErT to reprogram EMT-induced cells out of a dormant-like state, and to restore the transcriptional program promoting cell cycle progression (Fig. 3f). Reflective of this, LNCaP–iSnail cells induced into an EMT state were observed to have reduced proliferative capacity, which was restored with MErT (Fig. 3g). Confirming this phenotypic plasticity, LNCaP–iSnail cells held in an EMT state remained as individual invasive cells following seeding into 3D Matrigel^™^ cultures (Fig. 3h, bottom right panel). In contrast, cells allowed to undergo MErT had the capacity to re-initiate proliferation and form multi-cellular tumour spheroids (Fig. 3h, bottom left panel).

To examine metabolic effects driven by EMP, the XF24 Extracellular Flux Analyzer (Seahorse Biosciences) was used to measure the oxygen consumption rate (OCR) and extracellular acidification rate (ECAR) to quantify levels of oxidative phosphorylation (OXPHOS) and glycolysis, respectively. Induction of EMT for 1 and 5 days (E1 and E5) resulted in a dramatic decrease of both basal and maximal rates of OXPHOS and glycolysis (Figs. S4A and 3I), inducing a state of overall metabolic quiescence (Fig. 3i, lower left quadrant). This state was not observed following treatment of control LNCaP–iGFP cells with Dox over the same time periods (Figure S4A). MErT induced by the removal of Dox treatment for 10 days returned metabolic activity to pre-EMT levels (Figs. S4B and 3I) during the reactivation cell cycle progression. Of note, both basal and maximal levels of OXPHOS following MErT were significantly elevated compared to the levels detected in cells prior to EMT induction (Fig. 3i, top right quadrant and S4Bii). While more intensive studies are required, these results indicate that tumour cells that have passed through a reversible EMT may acquire an elevated capacity for OXPHOS. Collectively, these results provide a snapshot of the biological processes occurring during EMP and its direct effect on cell cycle, metabolism, and proliferation.

### MErT is enriched in metastatic prostate cancer

**Fig. 4:**
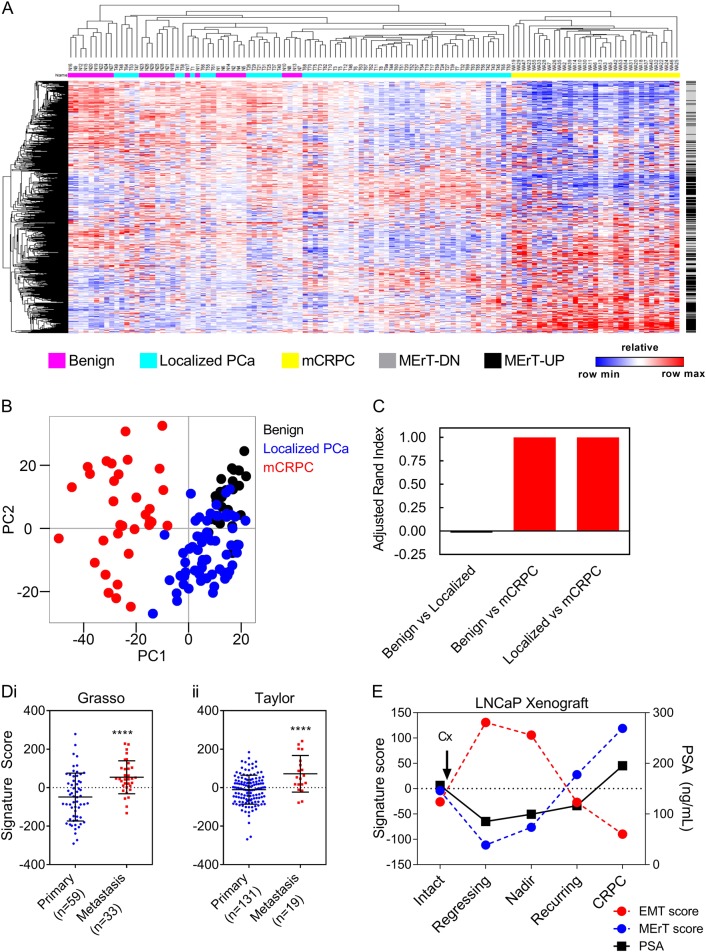
MErT is enriched in castrate-resistant prostate cancer (CRPC). **a** Unsupervised hierarchical clustering of 28 benign, 59 localised prostate cancer and 33 metastatic CRPC samples based on the expression of the MErT gene expression signature (MErT GES). Patient samples and expression data from the Grasso cohort [23] (GSE35988). MErT-DN / MErT-UP refers to downregulated / upregulated genes within the MErT GES. Heatmap color refers to normalised z-score. **b** Principal component analysis plot of the expression of the MErT GES in benign, localised PCa and mCRPC samples from the same cohort [23]. **c** Adjusted Rand Index of the MErT GES expression in benign vs localised PCa samples, benign vs mCRPC samples, and localised PCa vs mCRPC samples [23]. **d** Scatter plots showing the MErT GES score in samples of localised and metastatic PCa: **di**; Grasso cohort [23]; and **dii**; Taylor cohort [25]; (MSKCC; GSE21034). Error bars indicate SEM. **** *p* < 0.0001; unpaired *t*-test. **e** Enrichment scores of the EMT GES and MErT GES in LNCaP xenografts during PCa progression. Serum levels of prostate-specific antigen (PSA) are also shown

As EMT has been extensively studied in multiple human carcinoma types [17–22], we focused on examining the MErT process in prostate cancer. To generate a MErT gene expression signature (GES), we used GSEA to rank the expression of genes in the MErT20 samples as compared to the EMT5 samples (see Materials and methods section for detailed description). The top and bottom 500 ranked genes were selected to populate the signature (Supplementary dataset 2). Unsupervised hierarchical clustering (Fig. 4a) and principal component analysis (Fig. 4b) using the MErT GES in a University of Michigan dataset (Grasso) [23], identified localised PCa samples to cluster with benign prostate tissue samples, while all of the mCRPC samples formed a distinct cluster of their own (Fig. 4a, b). We also employed the adjusted rand index method (ARI) [24] to assess the pair-wise clustering power of the MErT GES (Fig. 4c). An ARI of 1 denotes perfect clustering and an ARI ≤ 0 indicates clustering not better than chance. It was observed that the MErT GSE separated mCRPC samples from benign and localised PCa samples with an ARI = 1, but was poor in separating benign from localised PCa samples (ARI = −0.017) (Fig. 4c). This suggests a distinct difference in the expression of the MErT GES between samples of mCRPC and benign/localised PCa samples. This was further supported by the positive and significant enrichment score of the MErT GES in mCRPC samples when compared to localised PCa samples from the Grasso cohort [23] (Fig. 4di) as well as in the Taylor cohort [25] (Memorial Sloan Kettering Cancer Centre; MSKCC; Fig. 4dii).

We then examined how EMP-associated gene expression is altered during the progression of androgen-dependent PCa to CRPC following ATT. As EMP incorporates both EMT and MErT, we generated an EMTGES derived using the methodology employed previously for the MErTGES, with the exception that the ranked list was generated using the expression data of EMT5 compared to No Dox (Supplementary dataset 2). The EMT and MErTGESs were then interrogated in the transcriptomes of LNCaP castrate-resistants collected from intact nude mice and during the progression of castrate-resistants to CRPC following castration of host animals (GSE44319) [26, 27]. PSA regressing and nadir castrate-resistants presented with a high EMT and a low MErT score, indicating that these tumours had enrichment of EMT-associated genes early after castration (Fig. 4e). In post-castrated PSA recurring tumours, scores for both GESs returned to similar levels as castrate-resistant tumours collected from non-castrated intact mice (Fig. 4e). Tumours progressing to CRPC were determined to have an elevated MErT score and a reduced EMT score as compared to castrate-resistant tumours from intact mice (Fig. 4e). This provides evidence that ATTs, in this case castration, can activate a global program of EMT in PCa castrate-resistant tumours, which is dynamically regulated during progression to CRPC via the reactivation of AR/androgen-signalling and promotion of MErT.

### MErT is not the antithesis of EMT—revealing a transcriptional memory of plasticity in mCRPC

**Fig. 5:**
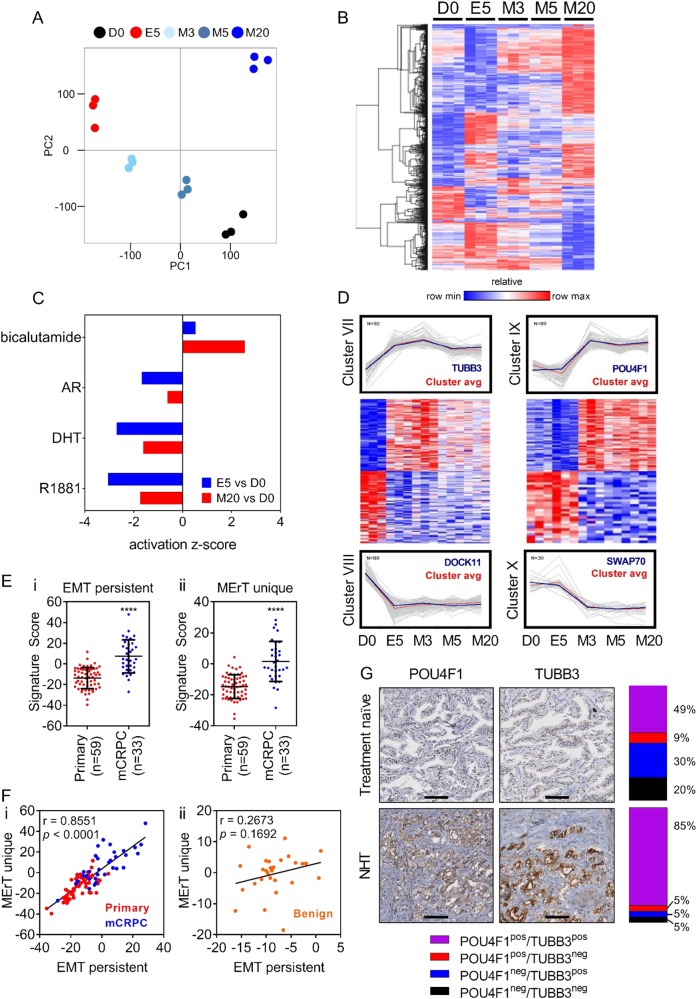
EMP transcriptional memory is enriched in metastatic castrate-resistant prostate cancer. **a** Principal component analysis as cells undergo transition from an epithelial (No Dox; D0) to a mesenchymal (EMT5; E5) and back to an epithelial (MErT; M3–20) state. **b** Heatmap showing differential transcript expression between MErT20 (M20) and No Dox (D0) (1564 gene identities can be found in the supplementary dataset 1). Heatmap color refers to normalised *z*-score. **c** Activation scores of upstream regulators in transcripts differential in EMT5 (E5) vs No Dox (D0) and MErT20 (M20) vs No Dox (D0). **d** Transcriptional clusters representing persistent transcript alterations. Cluster VII shows transcripts that remained upregulated and Cluster VIII shows transcripts that remained downregulated during the 20 day MErT. Cluster IX shows transcripts that were unaltered during EMT (E5) and then became newly activated by 3 days of MErT (M3) and remained for 5 and 20 days (M3,5–20). Cluster X shows transcripts that were unaltered during EMT (E5) and then became newly repressed by 3 days of MErT (M3) and remained for 5 and 20 days (M5–20). Cluster avg is the average transcriptional pattern of the transcript cluster. Heatmaps represent gene expression within Clusters VII and VIII, and within clusters IX and X. Heatmap color refers to normalised *z*-score. **e** Enrichment score of the (**ei**) EMT persistent (Clusters VII/VIII combined) and (**eii**) MErT unique (Clusters IX/X combined) signatures in samples of localised PCa and mCRPC from the Grasso dataset [23]. **f** Correlation plot of the EMT persistent and MErT unique signature scores in (**fi**) localised PCa and mCRPC samples (**fii**), and benign prostate tissue from the Grasso dataset. Error bars indicate SEM. **** *p* < 0.0001; unpaired *t*-test. **g** Immunohistochemical staining of POU4F1 and TUBB3 in samples from treatment naïve patients, and during neoadjuvant hormone therapy (NHT). Breakdown of patient numbers can be found in Table S3. Scale bars are 100 µm

Principal component analysis of the Snail-dataset identified a time-dependent connectivity pattern as cells reverted from a mesenchymal to an epithelial state (Fig. 5a). It was evident from the 2nd principal component (PC2) that LNCaP–iSnail cells following a 20 day MErT were transcriptionally altered in comparison to untreated parental epithelial cells (Fig. 5a). Indeed, examination of the transcript expression between the two groups (MErT20 vs No Dox; fold change ± 1.5 and *p* ≤ 0.05) identified 1564 differentially expressed probes (841 individual genes; Fig. 5b; Supplementary dataset 1). To gain insights on the potential biological functions altered between MErT20 and D0 cells, we analysed these 841 genes using Ingenuity Pathway Analysis^®^ (IPA^®^, Ingenuity Systems Inc.). IPA Upstream Regulator Analysis identified positive activation of gene networks regulated by the anti-androgen bicalutamide and a converse negative activation of gene networks regulated by the androgens dihydrotestosterone (DHT) and metribolone (R1881) (Fig. 5c). The 841 gene set was also significantly enriched in molecular networks related to “solid tumours,” “cell movement,” and “invasion of cells” (Figure S5). This suggests cells that have passed through a cycle of EMT/MErT may have dysregulation of the AR/androgen-signalling axis and retain biological phenotypes required for sustaining tumour growth and cancer metastasis.

As there are currently, no reported transcriptional signatures specific to the MErT process, we then focused on identifying a signature that becomes activated early during EMT/MErT and persists following a cycle of EMP. Four distinct clusters of transcripts that persisted in their differential expression were identified (Fig. 5d). Clusters VII and VIII contained transcripts significantly altered during EMT that persisted throughout 3, 5, and 20 days of MErT (cluster VII, *n* = 65 transcripts; cluster VIII, *n* = 54 transcripts; Supplementary dataset 1). Clusters IX and X contained transcripts not significantly altered during EMT, which then became altered in early MErT (3 days) and persisted through 5 and 20 days of MErT (cluster IX, *n* = 56 transcripts; cluster X, *n* = 22 transcripts; Supplementary dataset 1). To evaluate the clinical relevance of these novel transcriptional clusters, we assessed their expression in patient tumour samples from the Grass cohort 23. Transcripts within clusters VII and VIII (EMT persistent alterations), and clusters IX and X (MErT unique alterations) were combined and examined in localised PCa and mCRPC samples [23]. This revealed mCRPC samples to have increased enrichment of the “EMT persistent” and the “MErT unique” signatures, indicating more prominent expression of these transcriptional clusters in mCRPC than in localised PCa samples (Fig. 5ei, ii).

Having identified a potential transcriptional footprint of cancer cells that have experienced a reversible EMT in samples of mCRPC, the co-expression of these transcriptional clusters was examined. The “EMT persistent” and “MErT unique” signature scores had a strong positive correlation in both localised PCa and mCRPC samples (Pearson correlation, *r* = 0.86; *p* ≤ 0.0001; Fig. 5fi). Providing evidence for the co-expression/co-existence of these transcriptional clusters, or lack thereof in negative scoring samples, in primary and metastatic tumours. This correlation was not observed in benign prostate tissue samples (Fig. 5fii). TUBB3 and POU4F1 were then selected as sentinel biomarkers for the “EMT persistent” and “MErT unique” clusters, respectively, for validation using IHC in clinical PCa samples. To do this, IHC staining was performed for TUBB3 and POU4F1 on tissue microarrays (TMAs) containing radical prostatectomy (RP) samples from the Vancouver Prostate Centre Tissue Bank, including a unique subset of RP samples collected from men treated with neoadjuvant hormone therapy (NHT). Approximately half of the treatment naïve patient samples (49%) had co-expression of TUBB3/POU4F1 proteins (Fig. 5g). However, this increased to 85% in patients receiving NHT (Fig. 5g). Collectively, these results support the clinical relevance of these transcript clusters in mCRPC and their potential regulation by the androgen signalling axis that may serve as sentinel biomarkers for cells that have increased epithelial plasticity.

### A metastasis-derived plasticity signature expressed in primary tumour samples predicts poor patient prognosis

**Fig. 6:**
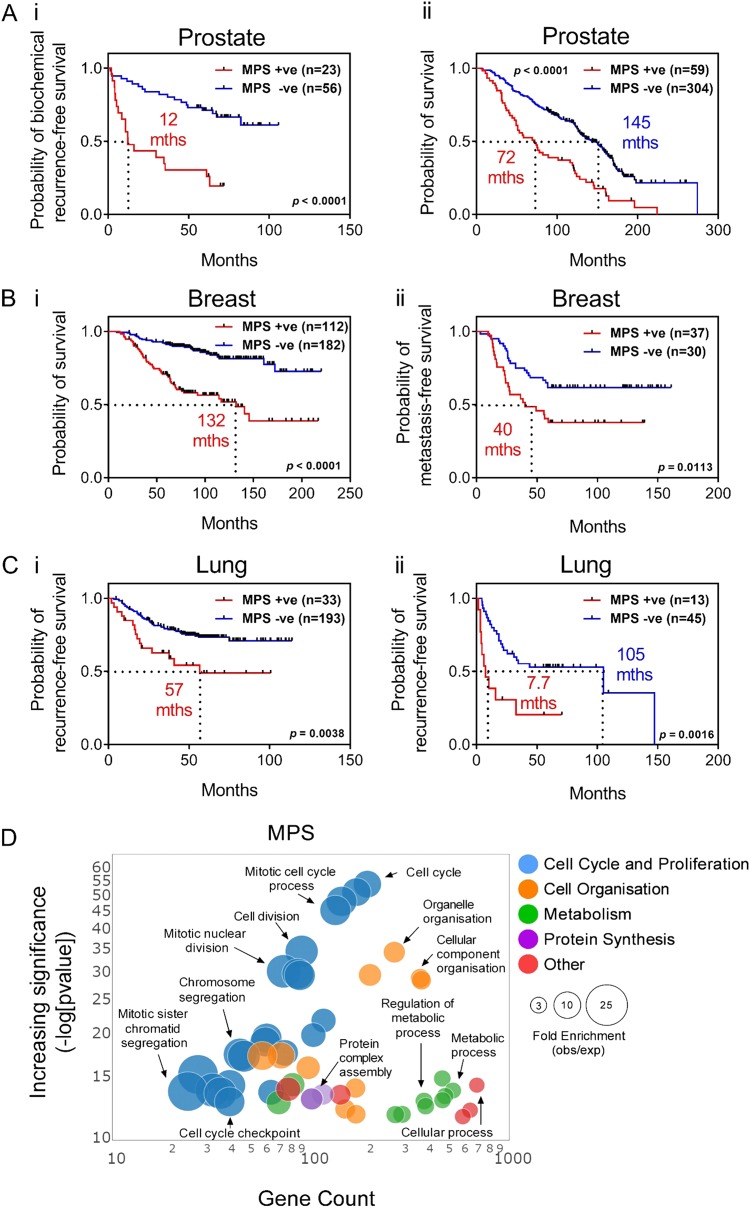
Snail metastatic plasticity signature (MPS) predicts poor patient outcome across multiple cancers. **a**–**c** Kaplan–Meier curves showing (**ai, ii**) prostate (Glinsky [32], Setlur [33]), (**bi, ii**) breast (van de Vijver [37], van’t Veer [38]), and (**ci, ii**) lung (Okayama [39], Lee [40]) cancer patient cohorts stratified according to a positive (+v, red line) or negative (-ve, blue line) MPS score. The *p*-values shown are from log-rank tests comparing the two Kaplan–Meier curves. **d** Bubble chart showing enriched gene ontology biological processes in the MPS

For this analysis we further refined the Snail-dataset to include only probes with significantly altered expression (fold change ± 1.5; *p* *≤* 0.05) at any time point during the EMT/MErT cycle (15,004 probes collapsed to 5732 genes; referred to as the EMPome dataset; supplementary dataset 1). Using GSEA, we interrogated the transcriptome of mCRPC samples and treatment naïve samples [23] with the EMPome dataset. This revealed 698 plasticity genes that were significantly elevated in samples of mCRPC compared to treatment naïve localised PCa samples (termed as the metastatic plasticity signature—MPS) (Supplementary dataset 2). To validate that the MPS was enriched in metastases, we examined a number of additional PCa patient cohorts containing metastatic samples [25, 28–31]. This identified the MPS to significantly overlap with gene signatures upregulated in metastatic samples across all of the patient cohorts examined (Figure S6). Concept analysis using the Oncomine™ database (www.oncomine.org) revealed MPS genes to be significantly associated (*p* *≤* 0.01, odds ratio [OR] ≥ 2.0) with not only metastatic PCa samples, but also in primary PCa samples from patients that developed recurrent disease (within 1–5 years) and poor survival (within 1–5 years) (Table 1). The stratification of patients based on a positive or negative MPS expression score revealed patients with MPS positive primary tumours to have a significantly shorter time to biochemical recurrence (Fig. 6ai; Glinsky cohort [32], *n* = 79; log rank *p* ≤ 0.0001; hazard ratio [HR]: 7.08) and shorter overall survival (Fig. 6aii; Setlur cohort [33], *n* = 363; log rank *p* ≤ 0.0001; HR: 3.27) compared to patients with primary tumours having MPS negative scores.

**Table 1:** Enrichment of the MPS in multiple prostate cancer patient cohorts

Dataset	Property	Overlap (no. of genes)	*p*-Value	Odds ratio	Top %
Taylor prostate 3	Metastatic status	287	4.38E-109	7.2	10
Varambally prostate	Metastatic status	238	6.14E-73	5.4	10
LaTulippe prostate	Metastatic status	107	2.40E-24	3.8	10
Yu prostate	Metastatic status	103	4.74E-22	3.6	10
Chandran prostate	Metastatic status	121	8.59E-14	2.3	10
Vanaja prostate	Metastatic status	128	2.44E-13	2.2	10
Lapointe prostate	Metastatic status	99	3.38E-13	2.5	10
Holzbeierlein prostate	Metastatic status	50	7.67E-08	2.5	10
Tamura prostate	Metastatic status	93	9.64E-08	1.9	10
Ramaswamy multi-cancer	Metastatic status	74	2.42E-06	1.9	10
Magee prostate	Metastatic status	21	0.003	2.1	5
Ramaswamy multi-cancer 2	Metastatic status	35	0.003	1.7	5
Glinsky prostate	Recurrence status at 3 years	93	2.94E-08	2	10
	Recurrence status at 5 years	90	2.32E-07	1.9	10
Nakagawa prostate	Recurrence status at 1 year	7	3.42E-04	7.5	5
	Recurrence status at 3 years	12	7.50E-06	7.4	10
	Recurrence status at 5 years	12	7.50E-06	7.4	10
	Survival status at 3 years	12	7.50E-06	7.4	10
	Survival status at 5 years	9	3.52E-06	12	5
Nakagawa prostate 2	Recurrence status at 1 year	7	8.37E-04	6.4	5
	Recurrence status at 3 years	12	3.94E-05	6	10
	Recurrence status at 5 years	7	8.37E-04	6.4	5
	Survival status at 3 years	13	5.95E-06	7	10
	Survival status at 5 years	10	0.001	4.3	10
Setlur prostate	Survival status at 1 year	46	2.28E-04	1.9	10
	Survival status at 3 years	46	8.94E-14	4.4	5
	Survival status at 5 years	50	1.84E-16	4.9	5
Taylor prostate 3	Recurrence status at 3 years	84	4.31E-14	2.8	5
	Recurrence status at 5 years	48	8.95E-27	9.1	1
Barwick prostate	Recurrence status at 5 years	7	3.42E-04	7.5	5

As many human carcinoma types share similar mechanisms of tumour progression, including evidence of EMT [19, 34–36], the prognostic ability of the MPS was assessed in additional human cancer types. As seen with the PCa patient cohorts, MPS genes were significantly (*p* ≤ 0.01, OR ≥ 2.0) associated with recurrent disease, time to metastasis, and poor overall survival across a number of carcinoma types, including breast, lung, kidney, and colon cancers (Supplementary dataset 2). For example, breast cancer patients (van de Vijver cohort [37], *n* = 294) with MPS positive primary tumour scores had a significantly shorter overall survival (Fig. 6bi; log rank *p* ≤ 0.0001; HR: 4.18) than patients with a MPS negative tumour score. In a separate cohort of breast cancer patients (van’t Veer cohort [38], *n* = 97), patients with MPS positive tumour scores had a shorter time to metastasis (Fig. 6bii, log rank *p* = 0.0113; HR: 2.22) than patients with MPS negative scoring tumours. Additionally, in two distinct cohorts of lung adenocarcinoma patients (Okayama cohort [39] *n* = 226; Lee cohort [40] *n* = 58), patients with MPS positive primary tumour scores also presented with shorter time to recurrence (Fig. 6ci; log rank = 0.0038; HR: 3.07; and Fig. 6cii; log rank = 0.0016; HR: 5.40).

Assessment of the biological processes enriched in the MPS revealed enrichment of cell cycle and proliferation processes, as well as cell organisation, metabolism, and protein synthesis (Fig. 6d). While cell cycle and proliferation processes were highly enriched, the association of MPS with poor clinical outcome was not solely dependent on intrinsic cell cycle/proliferative-associated gene expression as analysis conducted using a MPS devoid of genes annotated within the GO–BP category of “cell cycle” independently retained prognostic value (Figure S6). Taken together, these results not only implicate the MPS in poor clinical outcome, but also suggest that the cycling of epithelial and mesenchymal states may occur within primary tumours and is associated with poorer patient prognosis.

### Epithelial–mesenchymal plasticity associates with poor patient outcome

**Fig. 7:**
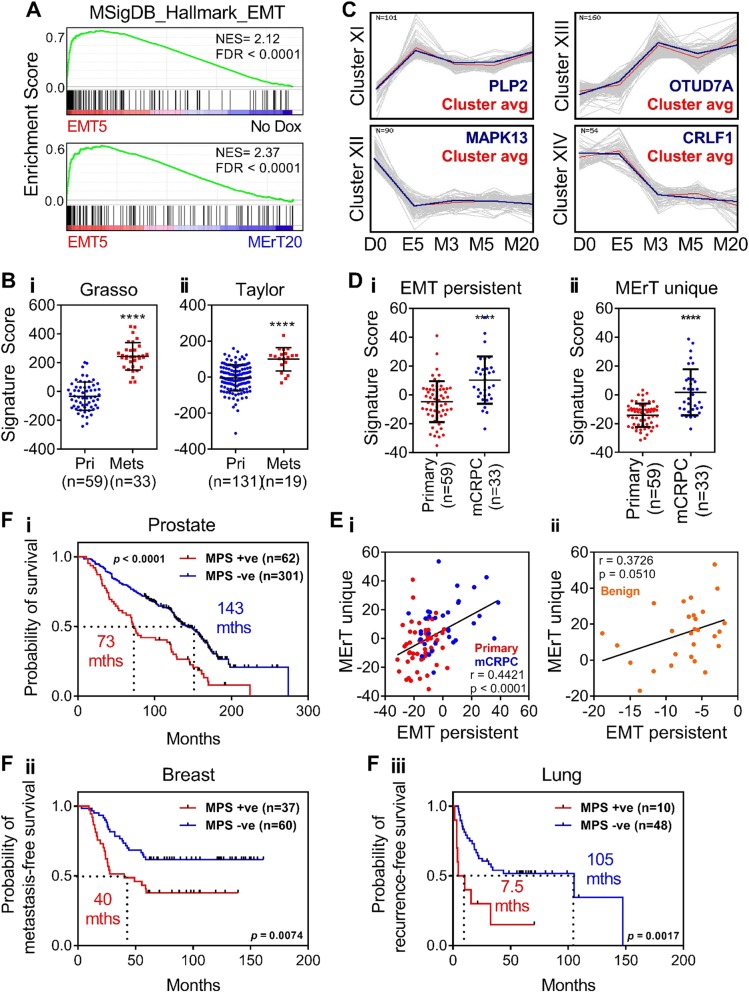
Validation of key concepts using the LNCaP–iSlug model. **a** GSEA enrichment plots of the “Hallmark” EMT signature (MSigDB [14]) within the transcriptional program regulated by 5 days of Dox treatment (EMT5), compared to untreated cells (No Dox; top panel), and compared to 20 days of Dox removal (MErT20). NES normalised enrichment score, FDR false discovery rate. **b** Scatter plots showing the Slug-derived MErTGES score in samples of localised and metastatic PCa from the **bi**; Grasso [23]; and **bii**; Taylor [25] datasets. Error bars indicate SEM. **** *p* < 0.0001; unpaired *t*-test. **c** Transcriptional clusters representing persistent transcript alterations. Cluster XI shows transcripts that remained upregulated and Cluster XII shows transcripts that remained downregulated during the 20 day MErT. Cluster XIII shows transcripts that were unaltered during EMT (E5) and then became newly activated by 3 days of MErT (M3) and remained for 5 and 20 days (M3, 5–20). Cluster XIV shows transcripts that were unaltered during EMT (E5) and then became newly repressed by 3 days of MErT (M3) and remained for 5 and 20 days (M5–20). Cluster avg is the average transcriptional pattern of the transcript cluster. **d** Enrichment score of the (**di**) EMT persistent (Clusters XI/XII combined) and (**dii**) MErT unique (Clusters XIII/XIV combined) signatures in samples of localised PCa and mCRPC from the Grasso dataset [23]. **e** Correlation plot of the EMT persistent and MErT unique signature scores in (**ei**) localised PCa and mCRPC samples (**eii**), and benign prostate tissue from the Grasso dataset. Error bars indicate SEM. ****, *p* < 0.0001; unpaired *t*-test. **f** Kaplan–Meier curves showing (**fi**) prostate (Setlur [33]), (**fii**) breast (van’t Veer [38]), and (**fiii**) lung (Lee [40]) cancer patient cohorts stratified according to a positive (+ve, red line) or negative (−ve, blue line) MPS score. The *p*-values shown are from log-rank tests comparing the two Kaplan–Meier curves

To examine whether our findings were specific to a Snail-induced EMT, we generated a SNAI2/Slug-driven reversible EMT model in LNCaP (LNCaP–iSlug; Figure S8) to validate a number of key concepts. Microarray profiling and analysis was performed using the same experimental setup and protocols as used for the LNCaP–iSnail model. GSEA examination of the transcriptional profile obtained following induction of Slug for 5 days had positive enrichment of genes within the “Hallmark_EMT” signature (MSigDB), compared to when Dox was removed for 20 days which had a negative enrichment (Fig. 7a). Similar to what was observed with the Snail-derived MErT signature, a Slug-derived MErT signature showed higher and significant enrichment in metastatic prostate cancer when compared to primary PCa (Fig. 7bi,ii). A Slug-induced EMT/MErT also contained EMT persistent and MErT unique gene signatures (Fig. 7c) that were found to have a higher and significant enrichment score in mCRPC than in primary PCa samples (Fig. 7di,ii). Similar to what was seen with the Snail-induced novel signatures, the signature scores of the Slug-induced novel signatures showed a significant and positive correlation in both primary PCa and mCRPC samples (Pearson correlation *r* = 0.44; *p* < 0.0001; Fig. 7ei). This also supported the co-expression of these novel signatures to be present in mCRPC samples compared to primary PCa samples where most samples had a negative score. Importantly, this correlation was not observed in benign prostate tissue collected from the same cohort (Fig. 7eii). Finally, we generated a Slug-derived MPS (430 genes), which was also found to associate positive scoring patients with significantly lower survival time, faster time to metastasis, and faster relapse when compared with negative scoring patients (Fig. 7fi, ii, iii; Oncomine concept analysis can be found in the supplementary dataset 2). Overall, the results obtained using the Slug-driven reversible EMT model support our findings generated in the Snail model.

## Discussion

The cycle of EMT and MErT (collectively termed epithelial–mesenchymal plasticity—EMP) represents a dynamic and complex set of phenotypic transitions that contribute, and are perhaps paramount, to cancer progression and therapy resistance. However, due to the transient nature of different states during EMP, its presence and role in patient tumour samples is constantly under debate as it is inherently difficult to identify tumour cells that have or are experiencing EMP. Furthermore, the classical markers used to confirm EMP, i.e. presence of E-cadherin, absence of vimentin, are not specific to the transition, and only describe the phenotypic state of the cell. Therefore, it is technically challenging to discern an epithelial tumour cell that has undergone EMT from one that may have been mesenchymal to begin with. On a similar note, markers such as vimentin are not specific to tumour cells as they are also expressed by other cell types in the tumour microenvironment, like, fibroblasts. Currently, to investigate whether EMP is relevant in cancer progression, research relies on experimental in vitro and in vivo models.

A number of in vivo studies have demonstrated that the inherent ability of cancer cells to shift phenotypic states facilitates metastasis. For instance, skin cancer cells induced into a mesenchymal state via expression of the EMT transcription factor Twist were not capable of forming overt metastasis unless loss of Twist expression allowed them to reverse back to their epithelial phenotype [12]. Similarly, intravenous injection of Prrx1 expressing breast cancer cells were more likely form metastases following loss of Prrx1 [10]. In PCa, studies by Celia-Terrassa demonstrated that while mesenchymal cancer cells were capable of escaping the primary tumour, metastatic outgrowth required cancer cells to be in an epithelial state [9]. Furthermore, mesenchymal cancer cells, as well as murine stromal tumour fibroblasts, have been shown to influence epithelial cancer cells to undergo EMT when in close proximity, bringing forward the hypothesis of possible collaboration between cancer cells in epithelial and mesenchymal states. Indeed, co-injection of hamster cheek pouch carcinoma cells that had undergone EMT and ones that hadn’t (non-EMT cells) into the bloodstream resulted in metastasis to more vascularly complex organs such as the liver or kidneys compared to injection of non-EMT cells alone, which metastasised to lymph nodes and bone [41]. Two recent studies using spontaneous models of metastasis with lineage tracing strategies highlight that an EMT-induced mesenchymal state evokes chemoresistant properties and promotes invasion from the primary tumour and into the vasculature (circulating tumour cells). However, metastases were commonly of an epithelial nature with only a small population of cancer cells identified to have experienced phenotypic plasticity at some stage during the metastatic process [42, 43]. Although the choice of EMT marker fibroblast-specific protein 1 (FSP1) to identify EMT in these studies was not able to capture all EMT events [44], both studies suggested inducing MErT as a therapeutic avenue to reverse EMT-induced chemoresistance [42, 43]. Taken together, there is accumulating evidence that cancer cell plasticity plays a role in facilitating cancer progression.

Our studies aimed to provide a more comprehensive transcriptional landscape of the complex gene expression events underpinning EMP and their association with clinical tumour samples. Using a PCa model of a reversible Snail-induced EMT (Fig. 1), we revealed the orchestrated and dynamic temporal transcriptional events that occur during the reversible transition of cancer cells between epithelial and mesenchymal states (Fig. 2). Analysis of the biological processes altered during the active transition of cells from an epithelial to a mesenchymal phenotype, combined with functional validation, revealed cells to enter a dormant-like and low-metabolically active state following EMT, which was relieved with MErT (Fig. 3). This is in agreement with speculations that EMT-induced dormant tumour cells need to reacquire their epithelial characteristics in order to regain their proliferative capacity [45, 46]. A number of studies have reported EMT-inducers to reduce cell division in a variety of tumour models [47–49], in which removal of EMT-inducers such as Twist [12] and Prrx1 [10] promoted metastatic growth. In addition, while the reprogramming of central metabolic pathways, such as oxidative phosphorylation (OXPHOS) and glycolysis, are “hallmarks” of cancer with well-established roles in regulating tumour growth and survival [50], it is only recently that metabolic reprogramming has been implicated in tumour invasion and metastasis using pre-clinical models [51, 52]. We have demonstrated that EMT-induced invasive cells enter a state of cell cycle arrest and metabolic quiescence, which is broken by MErT (Fig. 3).

Previously, the gene expression events associated with EMT reversion (i.e. MErT) in cancer cells have generally been regarded as the mirror image of those activated or repressed during EMT. In recent times, there has been a growing realisation that the MErT program may have unique features, or impart cells with stable phenotypic traits after passage through a reversible EMT [53, 54]. Through our profiling we identified a number of transcripts that were significantly altered in cells that had experienced a reversible EMT compared to cells that did not (Fig. 5). The identified transcripts had enrichment of processes supporting tumour growth but also activation of pathways associated with PCa progression in response to androgen suppression. Recent studies report that the dysregulation of AR signalling by inhibition of AR via ATTs influences tumour cell plasticity by activation of EMT-inducing markers and pathways [5–7, 55–58]. Within our dataset, gene networks regulated by AR as well as the androgens DHT and R1881, were inhibited by the transcriptional events occurring with EMT. In contrast, genes regulated by the anti-androgen, bicalutamide, were significantly activated, suggesting EMT to have a suppressive effect on the AR/androgen axis. Interestingly, this trend was maintained in transcriptional alterations that persisted following MErT as compared to non-induced parental cells.

Clinical analysis of PCa patient cohorts revealed that the transcriptional signature of MErT (MErT GES) was able to classify samples from mCRPC patients separately from primary PCa or benign samples (Fig. 4). Furthermore, the expression of the MErT GES was highly enriched in metastatic CRPC samples compared to primary PCa samples. To examine how EMP alters during PCa progression, we interrogated the transcriptomes from LNCaP xenografts mimicking PCa progression. This revealed the dynamic transcriptional plasticity taking place in response to castration and development into CRPC. Following castration the primary xenografts were enriched with an EMT transcriptional profile, which is in line with previous studies supporting that androgen targeted therapies induce an EMT [5–7, 55–58]. The enrichment of EMT peaked soon after castration (during tumour PSA regression), with a gradual return to pre-castration levels between PSA nadir and PSA recurrence. Progression into the CRPC stage was associated with the re-enrichment of the MErT profile, which was higher than pre-castration levels. Taken together, our data provide evidence that not only is tumour plasticity (or the balance between epithelial and mesenchymal states) regulated via the level of AR/androgen axis activity, but also that castrate-resistant plasticity itself can alter the expression of gene networks regulated by the AR/androgen axis.

It is recognised that the experimental MErT timeframe used in this study (20 days) may not be long enough for the reversion of some of these transcripts. One way we attempted to address this caveat, was to isolate transcripts that were persistent in their differential expression early in the experimental MErT timeframe (at 3 days) and persisted through 5 and 20 days. This identified the “EMT persistent” and “MErT unique” subprograms which were found to be significantly enriched in primary and metastatic CRPC samples. Furthermore, the expression of these two subprograms was highly correlated in primary PCa samples, with higher scores in mCRPC samples. Importantly, this correlation was lost in benign prostate tissue, supporting the occurrence of EMP in clinical tumour samples (Figs. 5, 7). These persistent transcripts have the potential to serve as a sentinel signature of cancer cells that have experienced a reversible EMT; however, further studies will be necessary to determine the resilience of these alterations over longer durations of MErT and/or during multiple EMT/MErT cycles. Previously, the inducible expression of Snail in human mammary epithelial cells was reported to bind transiently to its target promoters, triggering both transient and long-lasting chromatin alterations contributing to EMT [59]. Since EMT can evoke epigenetic changes in normal and malignant cell types [60], the integration of studies examining the dynamics of epigenetic alterations as cells cycle through EMT/MErT will likely provide novel insights into the persistent transcriptional alterations identified herein.

In general, EMT-derived gene signatures have been poor at predicting clinical outcomes of cancer patients. Despite the observation for the occurrence of EMT at the invasive front of multiple carcinoma types [61, 62], the EMT status of localised tumours has been unable to predict patient outcome [63, 64]. From our studies, a core set of plasticity genes (MPS) was found to be associated with poor prognosis when expressed in the primary tumours of prostate, breast, and lung cancer patients (Figs. 6, 7). Furthermore, it was demonstrated that this association was not solely dependent on intrinsic cell cycle/proliferation-related gene expression, a previously validated predictor of poor clinical outcome [15, 65], as a MPS devoid of these genes (MPS^CCR^) independently retained prognostic value similar to that of the MPS (Fig. 6 and S7). It was noted that the MPS^CCR^ was enriched in metabolism-related gene expression (Figure S7), suggesting that biomarker gene sets altered during the metabolic reprogramming of cancer cells may have clinical utility as novel predictors of patient outcome across multiple human cancer types. Moreover, these biomarker panels may aid in the identification of patients that may benefit from intervention with metabolic pathway targeted drugs [66] to slow disease progression and provide insights into new therapeutic strategies.

While the MPS will undoubtedly require further refinement and testing in extended patient cohorts, our data suggest that the cycling of epithelial and mesenchymal states via EMT/MErT may also occur at the primary tumour site (Figs. 6, 7). Specifically, the enrichment of the MPS was present in a subset of primary tumours within each patient cohort, and these subsets of patients had a shorter time to recurrence and reduced overall survival (Figs. 6, 7). Moreover, this was not specific to PCa patient cohorts, but broadly applicable across a number solid tumour types, including breast and lung carcinomas (Figs. 6, 7). The cycling of epithelial and mesenchymal states at the primary site was further supported by the significant correlation of the EMT persistent/MErT unique signatures observed in both primary and metastatic tumours, but not in benign prostate tissue samples (Figs. 5, 7). This is in contrast to the prevalent view that reversion of EMT occurs in cancer cells disseminated to distant tissue sites. Further work will be required to confirm the presence and prevalence of EMT/MErT cycling within primary tumours. However, we hypothesise that as EMT-induced invasive cells move throughout the tumour microenvironment and are exposed to varying spatial and paracrine cues from surrounding cells and tissue types, they may oscillate between epithelial and mesenchymal states more frequently than previously thought.

In summary, the results from this study lay the foundation for understanding the transcriptional landscape of epithelial–mesenchymal plasticity taking place during cancer progression. Importantly, this study identifies the potential of cancer cells cycling between epithelial and mesenchymal states within the primary tumour and this plasticity is significantly associated with poor patient outcomes. Additionally, the reawakening of EMT-induced dormant-like cells via MErT will need to be carefully considered for the application of potential anti-EMT therapeutic strategies. In particular, these treatments may have deleterious effects on metastatic tumour growth in patients with detectable circulating tumour cells or dormant occult metastases. Nevertheless, there is strong evidence that EMT confers chemoresistance, whereby the inhibition of EMT results in reduced development of chemoresistance and suppression of metastasis [42, 43]. We propose that the full spectrum of epithelial–mesenchymal plasticity needs to be considered for the application of new anti-metastatic therapies aimed at inhibiting EMT. A more effective approach may be to combine these therapies with agents targeting pathways arising from MErT that increase tumour cell survival and treatment resistance.

## Materials and methods

### Tissue culture and generation of EMP model

LNCaP (ATCC), Rockville, MD), LNCaP–iSnail, LNCaP–iSlug, and –iGFP cells were grown in phenol red free Roswell Park Memorial Institute—1640 medium (RPMI) (Thermofisher) containing 5% foetal bovine serum (FBS; ThermoFisher), 1% streptomycin –penicillin, and 0.05% gentamycin (Life Technologies) at 37 °C with 5% CO_2_. HEK293T (ATCC) cultured in Dulbecco’s modified Eagle’s medium (DMEM) supplemented with 10% heat inactivated FBS at 37 °C with 5% CO_2_. The pENTR223.1 entry clone containing the cDNA of SNAI1 (Snail) and eGFP were recombined into the pINDUCER20 lentiviral construct [13] using Gateway^®^ LR Clonase^®^ II enzyme mix according to manufacturer’s instructions (Thermofisher). Lentiviral packaging and supernatants were generated by transient transfection of HEK293T cells (ATCC) as described previously [67]. Stable LNCaP (ATCC) sublines expressing pINDUCER20-Snail, pINDUCER20-Slug, or pINDUCER20-GFP were generated by lentiviral transduction followed by selection with 1 mg/mL G418 (Invivogen). Optimal doxycycline hyclate (Dox; Sigma Aldrich) concentration for induction of cDNA expression was determined to be 1 μg/mL (data not shown) and was refreshed every 48 h of cell culture.

### Immunoblot analysis

Cells were harvested in 1x RIPA lysis buffer (Sigma Aldrich) containing protease inhibitor (Roche), phosphatase inhibitor cocktail 2 (Thermofisher) and 1 M NaF. Cell lysates were clarified by centrifugation at 21,130*g* for 10 min at 4 °C. Proteins were resolved using gradient (4–12%) NuPage gels (Thermofisher) and transferred onto nitrocellulose or Immobilon PVDF membrane (Millipore). Proteins were visualised using Immobilon Western Chemiluminescent HRP Substrate (Millipore) and a ChemiDoc Imager (Biorad). Information on antibodies and concentrations used can be found in Table S1.

### Immunofluorescence

Immunofluorescent staining of cells in monolayer and 3D Matrigel^™^ cultures was performed as previously described [67]. The antibodies used were rabbit anti-Snail (Cell Signalling Technology, Clone C15D3), rabbit anti-Slug (Cell Signalling Technology, Clone C19G7), mouse anti-E-cadherin (BD Biosciences, Clone 36), and mouse anti-vimentin (Sigma Aldrich, Clone V9). Secondary antibodies used were AlexaFluor® 568 goat anti-rabbit IgG and AlexaFluor® 488 goat anti-mouse IgG antibodies (Thermofisher).

### 3D Matrigel^™^ assays

The set-up and maintenance of 3D cultures using Matrigel™ (Corning) was performed as previously described [67]. Upon spheroid formation (10 days), Dox was added to induce Snail or GFP expression and refreshed every 2 days. For assessment of cell invasion, images were taken of all wells and the number of invasive protrusions per spheroid were manually counted. A minimum of 200 spheroids were counted for each treatment group at 2 and 4 days post-Dox treatment.

### qRT-PCR analysis

RNA was isolated using the Direct-zol™ RNA MiniPrep kit (ZymoResearch) and cDNA synthesised using Superscript III reverse transcriptase (Thermofisher) as per the manufacturer’s protocol. qRT-PCR was performed using SYBR Green Real-Time PCR master mix (Thermofisher) in conjunction with the Applied Biosystems 7900HT Fast Real-Time PCR System. PCR amplification was performed following an initial 10 min denaturation step at 95 °C with 40 cycles at 95 °C for 15 s and 60 °C for 60 s. Melt curve analysis was included in each run. Gene expression was quantified using the ^ΔΔ^Ct method relative to untreated cells. RPL32 levels were used to normalise cDNA loading. Primer sequences can be found in Table S2.

### Cell proliferation assay

LNCaP–iSnail cells were seeded at a density of 5 × 10^4^ cells per well in a 96-well plate with the indicated Dox treatment. Cell proliferation was assessed 5 days post seeding by imaging and enumeration of DAPI (Thermofisher) stained cells using the Cytell Cell Imaging System (GE Healthcare Life Sciences) at the indicated time points.

### Flow cytometry and cell cycle analysis

Cells were processed and DNA content analysed using flow cytometry as described previously [68]. The percentage of cells in each cell cycle phase was calculated with ModFit LT (Verity Software House) based on DNA histograms of 20,000 cells per treatment. For measuring GFP expression in LNCaP–iGFP cells treated with or without Dox, cells were harvested and immediately analyzed using a FC500 flow cytometer (Beckman Coulter) and data analyzed using FACS Express software (DE Novo Software).

### Seahorse xf24 extracellular flux assays

Oxygen consumption rate (OCR) and extracellular acidification rate (ECAR) were measured using Seahorse XF24 Extracellular Flux Analyzer (Seahorse Bioscience, USA). LNCaP–iGFP and –iSnail cells were maintained in RPMI with 5% FBS or treated for 5 days with Dox or 5 day in the presence of Dox, followed by 10 days in Dox-free medium. Each treatment was prepared to be ready on the day of analysis. One day prior to analysis, cells were seeded into 24-well Seahorse plates at 3 × 10^5^ cells per well with Dox treatments maintained, and an additional group receiving Dox upon seeding (Dox 1 Day). On the day of analysis, medium was replaced with basal assay medium comprised of unbuffered DMEM (Sigma, D5030) supplemented with glucose (11.1 mM), glutamax (2 mM), sodium pyruvate (1 mM); pH 7.4 for 1 h in non-CO_2_ incubator at 37 °C. OCR was measured in basal conditions and following sequential addition of 1.2 µM oligomycin (injection 1), 1 µM carbonyl cyanide-4-(trifluoromethoxy) phenylhydrazone (FCCP) (injection 2) and 1 µM each of antimycin A and rotenone (injection 3). ECAR was measured in basal conditions and following sequential addition of 10 mM glucose (injection 1), 1.2 µM oligomycin (injection 2) and 100 mM of 2-deoxyglucose (2-DG) (injection 3). At the end of the protocol, total DNA was analysed using the CyQuant DNA quantification kit (Thermofisher) against a standard curve of DNA from a known number of LNCaP cells. Oxygen consumption (pmol/min) and acidification rate (mpH/min) were normalised to cell number.

### Immunohistochemical staining of patient tissue microarray (TMAs)

Prostate cancer specimens were obtained from the Vancouver Prostate Centre Tissue Bank according to institutional guidelines. Specimens were examined by Hematoxylin and eosin staining and desired areas were marked on the paraffin blocks. TMAs were manually constructed (Beecher Instruments) by punching duplicate cores (1 mm) for each sample (Table S4). TMAs were stained with antibodies specific to POU4F1 (1:320 in Ventana Discovery antibody diluent; Polyclonal; Catalogue number: AB5945; Merck) or TUBB3 (1:100 in Ventana Discovery antibody diluent; Clone: TUJ1; #801201, Biolegend) using the Ventana Discover XT™ autostainer (Ventana Medical Systems) and scanned with a Leica SCN400 scanning system (Leica Microsystems). Due to the non-homogeneous nature of prostate cancer, scoring was performed manually by an experienced pathologist (Dr. Ladan Fazli). The scoring consisted of a four point scale: 0 = no staining of tumour cells; 1 = faint/focal or questionable staining; 2 = staining of convincing intensity in the majority of the tumour cells; and 3 = staining of strong intensity in the majority of tumour cells. Details on number of patients and staining categories can be found in Table S3.

### Microarray gene expression profiling and analysis

RNA for each condition was collected at the indicated time points (No Dox, EMT5, MErT3, MErT5 and MErT20) in biological triplicates and the quality was analyzed using a Bioanalyzer (Agilent). RNA was then prepared for microarray profiling as described previously using a custom 180 K Agilent array platform (Agilent-027516 VPC Human 180 K v2; GPL14873) [69]. Microarray raw data were processed using the Agilent Feature Extraction Software (v10.7) as described previously [69]. Differential expression was determined using a Bayesian adjusted t-statistic from a Linear Models for Microarray Data (LIMMA) linear model. As the 180 K array covers both ref-seq and non ref-seq genes, for our analyses we only used the probes covering ref-seq genes. The gene expression data have been submitted to Gene Expression Omnibus (GEO) with the accession number GSE80042. Gene expression was considered significant if fold change was +/-1.5 and *p* ≤ 0.05 (adjusted for a false discovery rate (FDR) of 5%). Prior to derivation of EMT and MErT signatures, all probes significantly (fold +/−1.5; *p* ≤ 0.05) altered by Dox treatment, as compared to non-treatment groups, in the LNCaP–iGFP model at any time point were removed from further analysis (Snail-dataset).

The filtered gene lists were examined by Ingenuity Pathway Analysis® (IPA) for functional annotation and gene network analysis. Gene Set Enrichment Analysis (GSEA; http://www.broad.mit.edu/gsea) was used to identify enrichment of gene signatures contained in the Molecular Signatures Database (MSigDB). Gene set permutation analysis was performed using the “weighted” or “classic” enrichment statistic and the signal-to-noise metric for gene ranking. Transcript clusters were identified using the pattern matching function within The National Institute on Aging (NIA) Array analysis tools (http://lgsun.grc.nia.nih.gov/ANOVA/). Rand Index analysis was performed as previously described [24]. The microarray data were uploaded to Oncomine™ v4.5 (www.oncomine.com) and overlaid with published microarray datasets using the Concept Analysis tool. A number of datasets from the Oncomine™ concept analysis table were selected for testing the prognostic qualities of the MPS. The examined datasets were selected based on having adequate sample size (patient size over 50) and access to the patient outcome details (including time to event). Signature scoring was performed as previously described [16].

### Derivation of MErT gene expression signature (GES)

GSEA was used to generate a ranked list of genes using the Signal2Noise ranking metric (descending order) in the expression data of MErT20 as compared to EMT5. The top and bottom 500 ranked genes were selected to generate the MErT signature.

### Derivation of emt gene expression signature (ges)

GSEA was used to generate a ranked list of genes using the Signal2Noise ranking metric (descending order) in the expression data of EMT5 as compared to No Dox. The top and bottom 500 ranked genes were selected to generate the EMT signature.

### Statistical analysis

All experiments were conducted in biological triplicates. Significance was determined by one-way, two-way ANOVA, or unpaired two-tailed Student’s *t*-test where appropriate. A *p* ≤ 0.05 was considered statistically significant. For survival analysis, Kaplan–Meier curves were drawn and differences between the curves were calculated by the log-rank test using GraphPad Prism Software (version 7.03), whereby *p* ≤ 0.05 was considered statistically significant. Fishers test was used to determine enrichment of gene signatures in metastatic samples from PCa patient cohorts (GSE3325 [30], GSE21034 [25], GSE68882 [29], GSE6752 [28], and GSE6919 [31] were downloaded from GEO). GSEA analysis generated a nominal *p*-value and FDR, reflecting the significance of geneset enrichment, estimated using a geneset-based permutation test. A *p* ≤ 0.05 and false discovery rate (FDR) of 5% was considered significant.

### Accession numbers

Raw microarray data is available from the Gene Expression Omnibus (GEO: GSE80042).

## Electronic supplementary material

Supplemental dataset 1 - Clusters

Supplemental dataset 2 - Signature lists and Oncomine

Supplemental Figures Tables and Legends

Video S1. LNCaP-iSnail cells grow to form non-invasive multicellular spheroids

Video S2. Pre-formed LNCaP-iSnail spheroids become invasive following induction of EMT

Table S4
