# Correction: A molecular portrait of epithelial–mesenchymal plasticity in prostate cancer associated with clinical outcome

**DOI:** 10.1038/s41388-018-0587-3

**Published:** 2018-12-03

**Authors:** Nataly Stylianou, Melanie L. Lehman, Chenwei Wang, Atefeh Taherian Fard, Anja Rockstroh, Ladan Fazli, Lidija Jovanovic, Micheal Ward, Martin C. Sadowski, Abhishek S. Kashyap, Ralph Buttyan, Martin E. Gleave, Thomas F. Westbrook, Elizabeth D. Williams, Jennifer H. Gunter, Colleen C. Nelson, Brett G. Hollier

**Affiliations:** 1 Australian Prostate Cancer Research Centre—Queensland, Institute of Health and Biomedical Innovation, Faculty of Health, School of Biomedical Sciences, Queensland University of Technology, Princess Alexandra Hospital, Translational Research Institute, Brisbane, QLD Australia; 2 Vancouver Prostate Centre, Department of Urologic Sciences, University of British Columbia, Vancouver, Canada; 3 Glycation and Diabetic Complications Group, Mater Research Institute, Translational Research Institute, School of Medicine, University of Queensland, Brisbane, QLD Australia; 4 Tissue Repair and Regeneration Program, Institute of Health and Biomedical Innovation, Queensland University of Technology, Brisbane, QLD Australia; 5 Verna and Marrs McLean Department of Biochemistry and Molecular Biology, Baylor College of Medicine, Houston, TX USA

Correction to: *Oncogene*; 10.1038/s41388-018-0488-5; published online 07 September 2018.

Following the publication of the above article, the authors noted an error in Fig. 4, panel B. The colours of the localized and mCRPC samples were accidentally switched. The authors have corrected the colour scheme and added a key to the figure. They have also updated the colour scheme of panel C, both bars are now red instead of one red and one blue. The authors wish to apologize for any inconvenience caused.

