# Supplementary material for: A molecular portrait of epithelial–mesenchymal plasticity in prostate cancer associated with clinical outcome
Source: Oncogene. 2018 Sep 7;38(7):913–34. doi: 10.1038/s41388-018-0488-5 (PMC6514858; doi:10.1038/s41388-018-0488-5)
Supplement: Supplementary file 3 — Supplemental Figures Tables and Legends [file 41388_2018_488_MOESM3_ESM.docx]

**
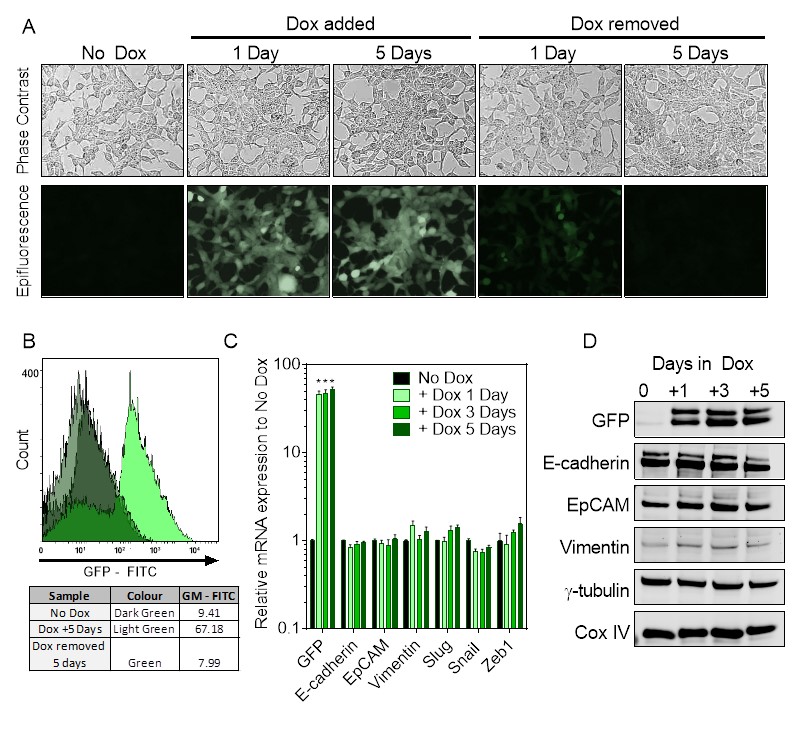
Supplemental Figures and Legends:**

Figure S1. Assessment of EMT-related markers in the LNCaP-iGFP cell model.

(A) Phase contrast and epifluorescent images of untreated LNCaP-iGFP cells, and cells treated with dox for 1 and 5 days, followed by the removal of Dox for 1 and 5 days. (B) GFP intensity was measured using flow cytometry for untreated LNCaP-iGFP cells, cells treated with dox for 5 days and then following Dox removal for 5 days. (C) Gene expression of *GFP*, *E-cadherin, EpCAM, Vimentin, Slug, Snail,* and *Zeb1* in LNCaP-iGFP cells treated with Dox for 1, 3, and 5 days. Gene expression was normalized to *RPL32* levels. Fold change is relative to untreated LNCaP-iGFP cells. Error bars indicate SEM for biological triplicates. One-way ANOVA, *p*‑value: * ≤ 0.05. (D) Western blot showing the expression of GFP, E-cadherin, EpCAM, and Vimentin proteins in untreated LNCaP-iGFP cells, and cells treated with Dox for 1, 3 and 5 days. γ-tubulin and Cox IV proteins were visualized for loading control purposes.

**
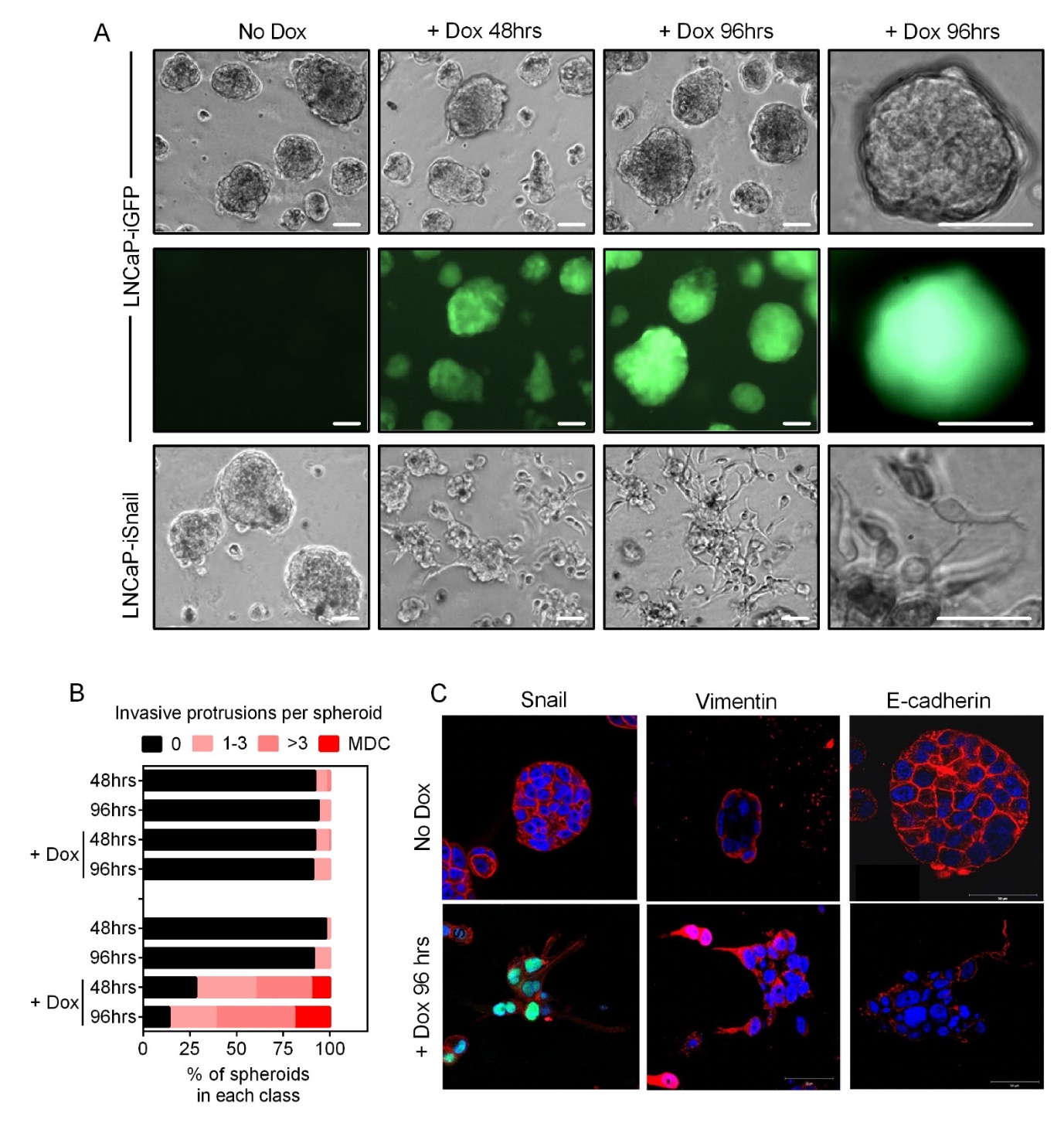
**

**Figure S2. Induction of LNCaP-iSnail tumor spheroid invasion following Dox-mediated Snail expression.**

LNCaP-iSnail and LNCaP-iGFP models were grown in modified 3D‑on‑top Matrigel™ assays for 10 days to form multicellular spheroids and then treated with dox for 4 days. **(A)** Representative phase contrast and epifluroescent images are shown over the 4-day dox treatment. Scale bars indicate 50 μm. **(B)** Bar chart showing the percentage of invasive colonies in each category for the indicated times. Each category described the invasive status of the spheroids: Black = absence of invasive cells, Light Pink = 1-3 protrusions, Pink = more than 3 protrusions, Red = multiple dissociated cells (MDC). (**C)** Immunofluorescence images showing the expression of Snail, E-cadherin, and Vimentin proteins in untreated and 4-day dox treated LNCaP-iSnail spheroids. Nuclei were visualized with DAPI. Scale bars indicate 50 μm.

**
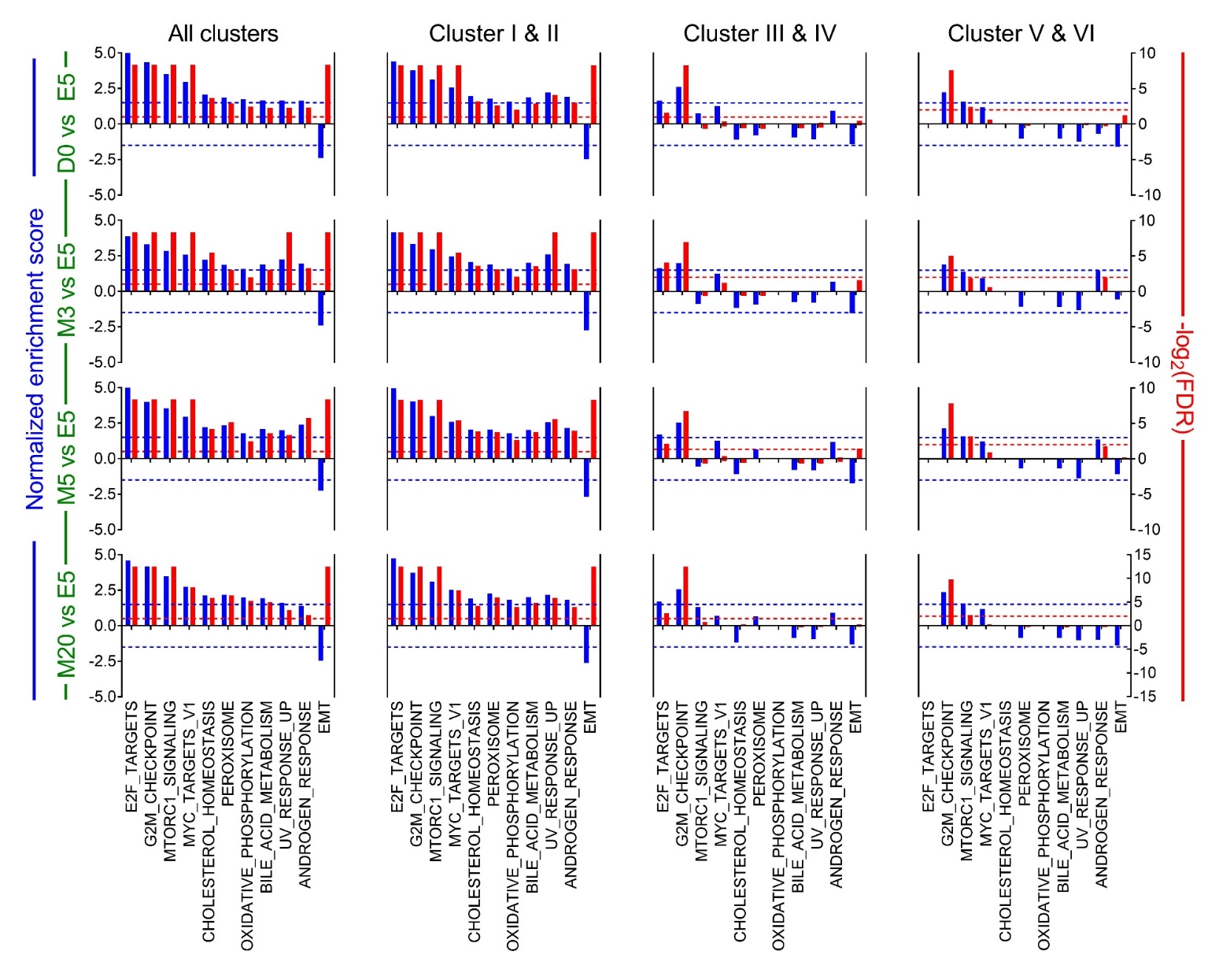
**

**Figure S3. Dynamic enrichment of biological processes during epithelial-mesenchymal plasticity.**

(**A**) GSEA was used to assess the enrichment of the “Hallmark” gene sets from the Molecular Signatures Database within each MErT transcriptional cluster (as compared to EMT5; E5), at the indicated time points (green). Blue dotted line indicates a normalized enrichment score ± 1.5; Red dotted line indicates a false discovery rate (FDR) of 0.25.

**
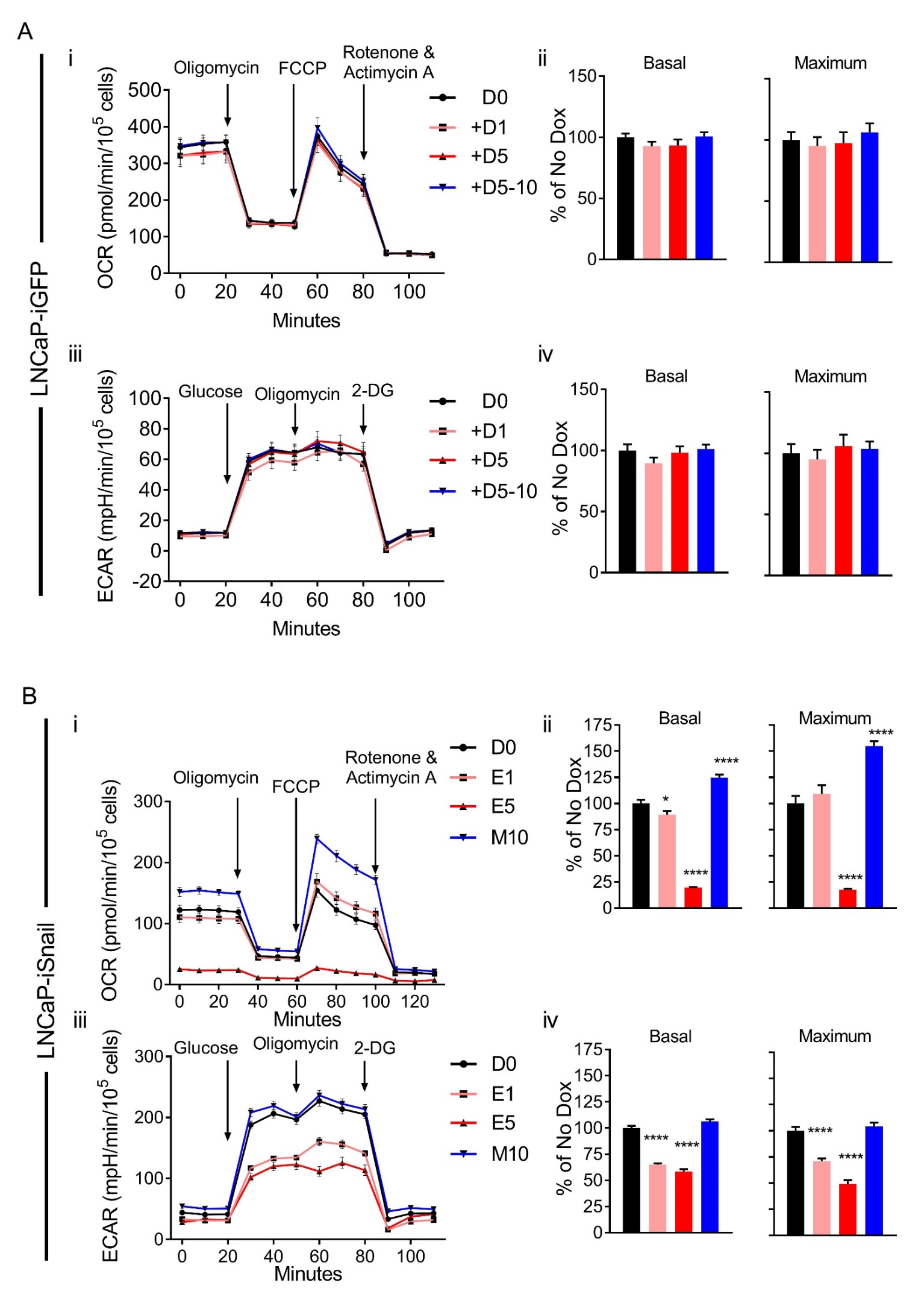
**

**Figure S4: Metabolic activity of LNCaP-iSnail and LNCaP-iGFP cells during dox**

(**Ai**) Measure of basal oxygen consumption rate (OCR) in LNCaP-iGFP cells treated with Dox 1 and 5 days (+D1, +D5) followed by removal for 10 days (+D5-10). . (**Aii**) Bar chart representation showing the basal and maximum respiration capacity relative to untreated LNCaP-iGFP cells (D0). Error bars indicate SEM of biological triplicates. FCCP: carbonyl cyanide 4-(trifluoromethoxy) phenylhydrazone. (**Aiii**) Measure of extracellular acidification rate (ECAR) in LNCaP-iGFP cells treated with Dox 1 and 5 days (+D1, +D5) followed by removal for 10 days (+D5-10). (**Aiv**) Bar chart representation showing the basal and maximum glycolytic capacity relative to untreated LNCaP-iGFP cells (D0). Error bars indicate SEM of biological triplicates. 2-DG: 2-deoxyglucose. (**Bi-iv**) Same measurements for the LNCaP-iSnail cells. D0: No Dox; E1: Treated with Dox for 1 day; E5: Treated with Dox for 5 days; M10: Treated with Dox for 5 days and subsequently removed for 10 days. One-way ANOVA; *p*-value: ****<0.0001.

**
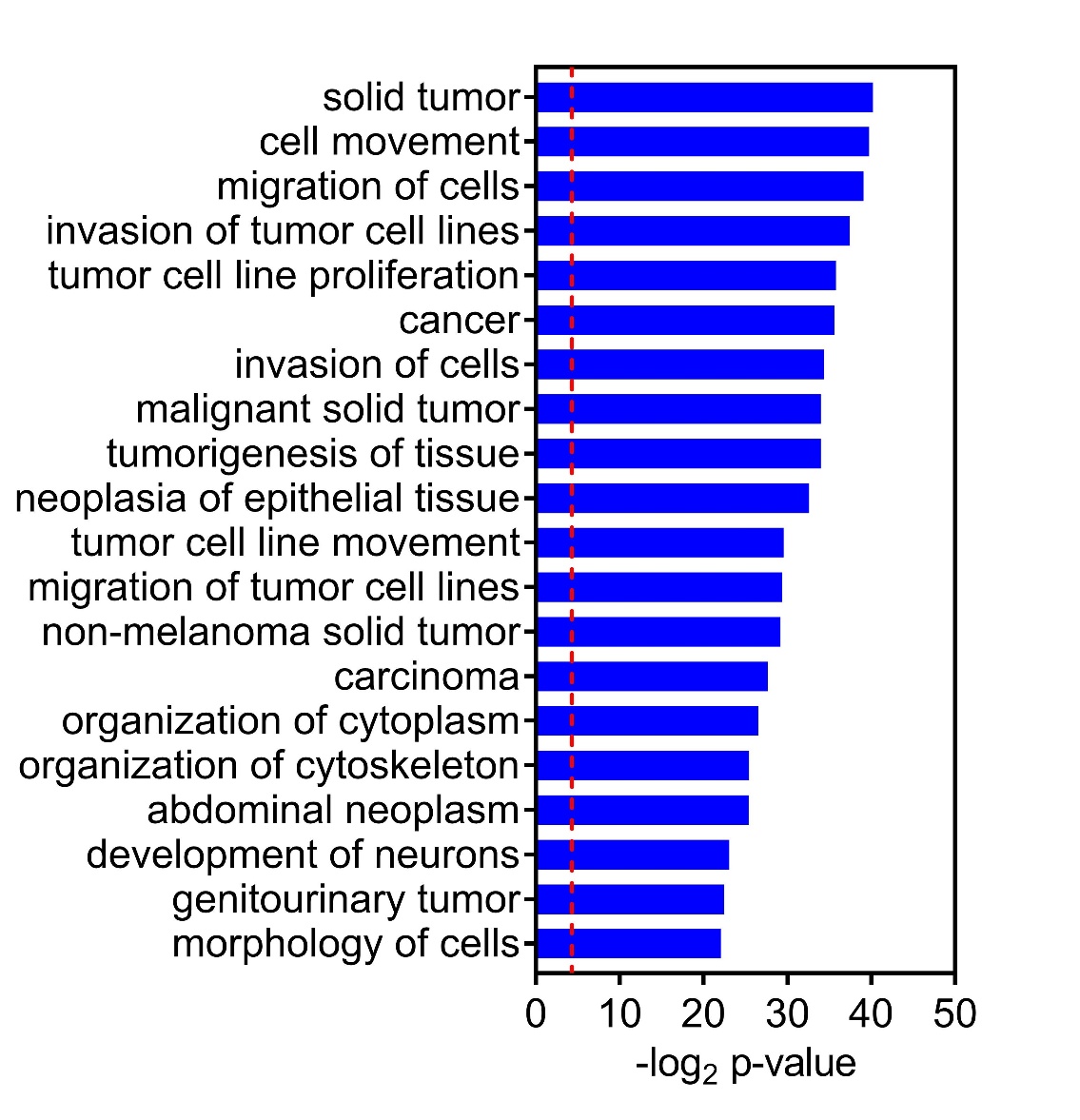
**

**Figure S5: Biological processes enriched in MErT20 compared to No Dox.**

Ingenuity Pathway Analysis was used to assess the enrichment of functions and disease in genes differential in MErT20 compared to No Dox.

**
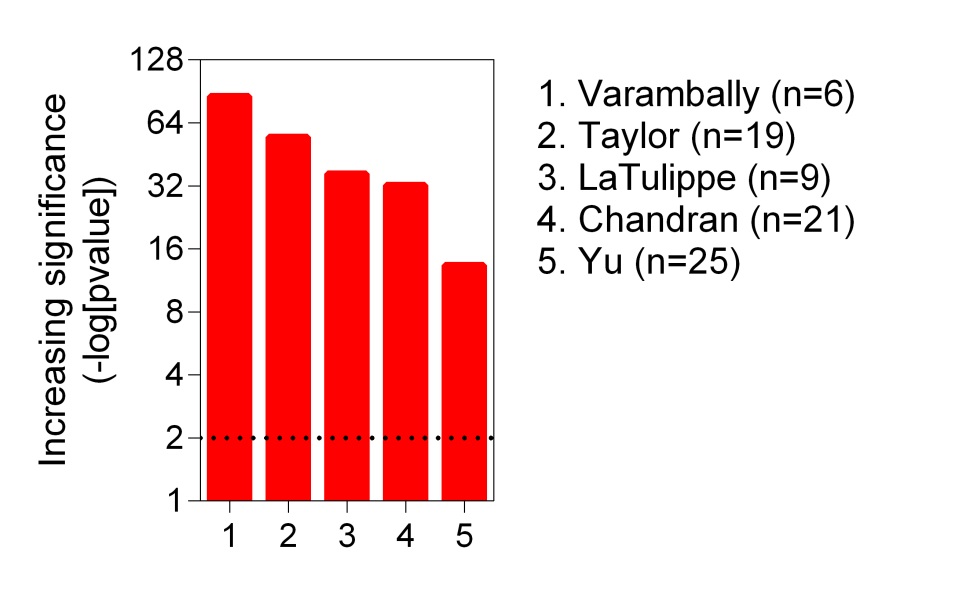
**

**Figure S6. Expression of the Metastatic Plasticity Signature (MPS) in prostate cancer datasets.**

The Metastatic Plasticity Signature (MPS) overlaps with genes significantly overexpressed (1.5 fold change; p<0.05) in metastatic samples as compared to localized PCa samples across multiple patient cohorts. Datasets examined (GSE3325(30), GSE21034(25), GSE68882(29), GSE6752(28), and GSE6919(31). *Dotted line* indicates *p* <0.01. n = number of metastatic samples in each dataset.

**
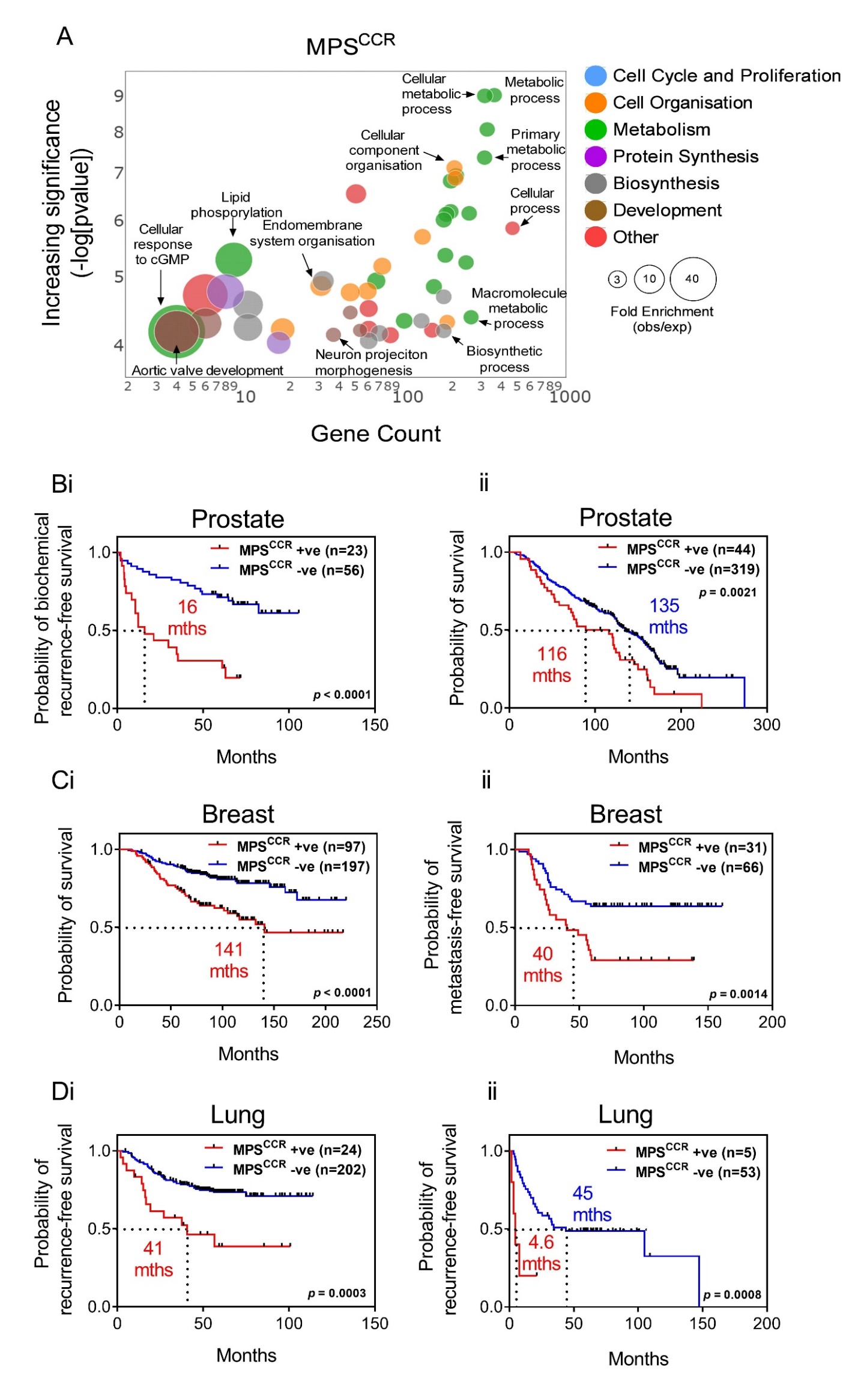
**

**Figure S7. The Metastatic Plasticity Signature (MPS) devoid of cell cycle genes (MPS^CCR^) predicts poor patient outcome across multiple cancers.**

(**A**) Bubble chart showing enriched gene ontology biological processes in the MPS^CCR^.
 (**B** to **D**) Kaplan-Meier curves showing (**Bi-ii**) prostate (Glinsky(32), Setlur(33)) , (**Ci-ii**) breast (van de Vijver(37), van’t Veer(38), and (D**i-ii**) lung (Okayama(39), Lee(40)) cancer patient cohorts stratified according to a positive (+ve; red line) or negative (-ve; blue line) MPS^CCR^ score. The *p*-values shown are from log-rank tests comparing the two Kaplan-Meier curves.


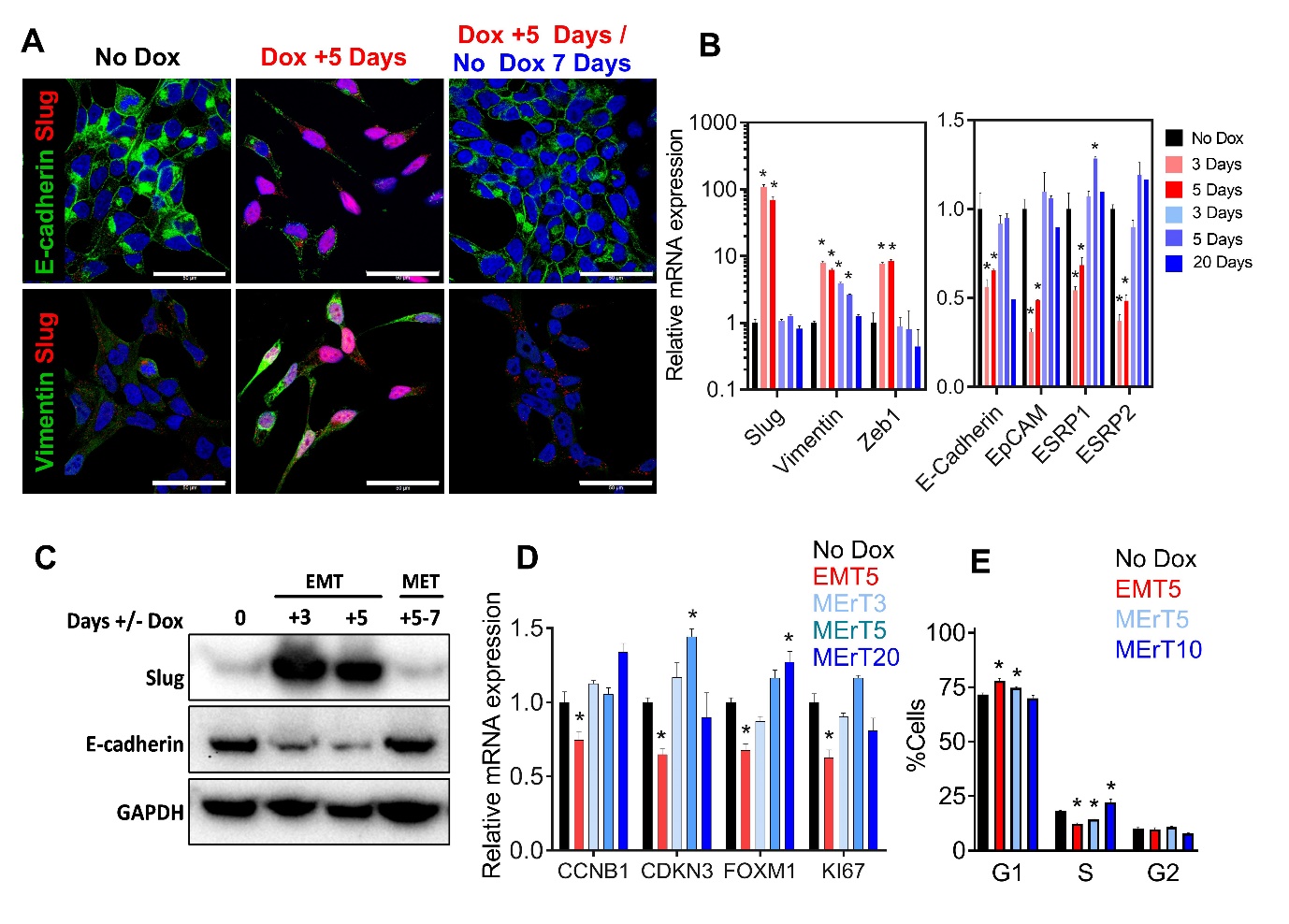


**Figure S8. Characterization of the LNCaP-iSlug model.**(**A**) Representative immunofluorescence images of Slug, E-cadherin, and vimentin, in untreated LNCaP-iSlug cells and treated with dox for 5 days with removal for 7 days. Nuclei were visualized using DAPI. Scale bars = 50 µm. (**B**) Gene expression of *Slug*, *vimentin*, *Zeb1*, *E-cadherin*, *EpCAM*, *ESRP1*, and *ESRP2* in LNCaP-iSlug cells treated with Dox for 3 and 5 days, followed by removal for 3, 5, and 20 days. Gene expression was normalized to RPL32. Fold change is relative to untreated cells. One-way ANOVA, *p*-value: *< 0.05. Error bars indicate SEM for triplicates. (**C**) Western blot showing expression of Slug and E-cadherin proteins in untreated LNCaP-iSlug cells, and treated with Dox for 3 and 5 days followed by removal for 7 days. GAPDH was visualized for loading control purposes. (**D**) Gene expression of *CCNB1*, *CDKN3*, *FOXM1*, and *Ki67* in LNCaP-iSlug cells treated with Dox for 5 days (EMT5), followed by removal for 3, 5, and 20 days (MErT3-20). Gene expression was normalized to RPL32. Fold change is relative to untreated cells. One-way ANOVA, *p*-value: *< 0.05. Error bars indicate SEM for triplicates. (**E**) Flow cytometry cell cycle analysis of untreated LNCaP-iSlug cells, and cells treated with dox for 5 days (EMT5) and subsequently removed for 5 and 10 days (MErT5-10). One-way ANOVA, *p*-value: *< 0.05. Error bars indicate SEM for biological triplicates.

**Video S1. LNCaP-iSnail cells grow to form non-invasive multicellular spheroids.**
 LNCaP-iSnail cells were grown in 3D Matrigel™ cultures for 14 days. Video captures days 10 to 14.

**Video S2. Pre-formed LNCaP-iSnail spheroids become invasive following induction of EMT.**
 LNCaP-iSnail cells were grown in 3D Matrigel™ assays for 10 days to form multicellular spheroids prior to treatment with Dox for 4 days. Video captures days 10 to 14.

**Supplemental Tables:**

**Table S1: Antibody specifications**

| **Antigen** | **Clone** | **Species** | **Dilution (of stock)** | **Catalogue #** | **Company** |
| --- | --- | --- | --- | --- | --- |
| Snail | C15D3 | Rabbit | 1:5000 | #3879 | Cell Signaling Technology |
| Slug | C19G7 | Rabbit | 1:1000 | #9585 | Cell Signaling Technology |
| GFP | D5.1 | Rabbit | 1:1000 | #2956 | Cell Signaling Technology |
| E-cadherin | 36 | Mouse | 1:2500 | 610181 | BD Biosciences |
| Vimentin | V9 | Mouse | 1:500 | V6630 | Sigma-Aldrich |
| EpCAM | D1B3 | Rabbit | 1:1000 | #2626 | Cell Signaling Technology |
| GAPDH | GAPDH-71.1 | Mouse | 1:10000 | G8795-100UL | Sigma-Aldrich |
| COXIV | 3E11 | Rabbit | 1:5000 | #4850 | Cell Signaling Technology |
| γ-tubulin | GTU-88 | Mouse | 1:4000 | T6557 | Sigma-Aldrich |

**Table S2: Primer sequences**

| Forward |  | Reverse |  |
| --- | --- | --- | --- |
| CCNB1 | AGAGCCATCCTAATTGACTG | CCNB1 | CAACCAGCTGCAGCATCTTC |
| CDKN3 | GGAAGAGCTTACAACCTGCC | CDKN3 | ACAGGTATAGTAGGAGACAAGC |
| FOXM1 | CTCCTTCTGGACCATTCACC | FOXM1 | CCAAGTGCTCGGGCAATTGT |
| MKI67 | CAAATTACAAGACTCGGTCCCTG | MKI67 | GGGAGGTCTTCATGGGCTTC |
| CTGF | CCTGGTCCAGACCACAGAGT | CTGF | TGGAGATTTTGGGAGTACGG |
| ZEB1 | CAACTACGGTCAGCCCT | ZEB1 | GCGGTGTAGAATCAGAGTC |
| CDH1 | TGCCCAGAAAATGAAAAAGG | CDH1 | GTGTATGTGGCAATGCGTTC |
| SNAI1 | CCTCCCTGTCAGATGAGGAC | SNAI1 | CCAGGCTGAGGTATTCCTTG |
| SNAI2 | GGGGAGAAGCCTTTTTCTTG | SNAI2 | TCCTCATGTTTGTGCAGGAG |
| VIM | GAGAACTTTGCCGTTGAAGC | VIM | GCTTCCTGTAGGTGGCAATC |
| EPCAM | TGCTCAAAGCTGGCTGCCAAATG | EPCAM | GTGCCGTTGCACTGCTTGGC |
| ESRP1 | TCCTGCTGTTCTGGAAAGTCG | ESRP1 | TCCGGTCTAACTAGCACTTCGTG |
| ESRP2 | GGGTCTGGGAAGTCAAGACAATG | ESRP2 | CTTCGAAAACAATTGACTGCTGG |
| RPL32 | GCACCAGTCAGACCGATATG | RPL32 | ACTGGGCAGCATGTGCTTTG |

**Table S3: TUBB3/POU4F1 staining information for patients on neoadjuvant hormone therapy and for treatment naïve patients**

| **On neoadjuvant hormone therapy (NHT)** | |  |
| --- | --- | --- |
| Stain | number of patient cores | % |
| TUBB3^pos^/POU4F1^pos^ | 97 | 85.1 |
| TUBB3^neg^/POU4F1^pos^ | 5 | 4.4 |
| TUBB3^pos^/POU4F1^neg^ | 6 | 5.3 |
| TUBB3^neg^/POU4F1^neg^ | 6 | 5.3 |
| Total | 114 | 100 |
|  |  |  |
| **Treatment naïve** |  |  |
| Stain | number of patient cores | % |
| TUBB3^pos^/POU4F1^pos^ | 226 | 49.2 |
| TUBB3^neg^/POU4F1^pos^ | 40 | 8.7 |
| TUBB3^pos^/POU4F1^neg^ | 138 | 30.1 |
| TUBB3^neg^/POU4F1^neg^ | 55 | 12 |
| Total | 459 | 100 |
