# Supplementary material for: A molecular portrait of epithelial–mesenchymal plasticity in prostate cancer associated with clinical outcome
Source: Oncogene. 2018 Sep 7;38(7):913–34. doi: 10.1038/s41388-018-0488-5 (PMC6514858; doi:10.1038/s41388-018-0488-5)
Supplement: Supplementary file 6 — Table S4 [file 41388_2018_488_MOESM6_ESM.docx]

**Table S4: List of TMAs**

| **TMA/ Human** | **# Patients** | **Patient information** |
| --- | --- | --- |
| Gleason-2009 1TMA | 88 | Untreated Radical prostatectomies, arrayed by Gleason grade, including adjacent benign |
| NHT 2009 | 88 | Radical prostatectomies with prior neoadjuvant therapy from 1-12 months |
| Survival-2011 (2 TMAs) | 176 | TMAs arrayed based on with or without LN metastasis |
| CRPC/ NEPC | 40 | CRPC/ TURP specimen including 12 NEPC |
